# A review of public acceptance of nature-based solutions: The ‘why’, ‘when’, and ‘how’ of success for disaster risk reduction measures

**DOI:** 10.1007/s13280-021-01502-4

**Published:** 2021-02-19

**Authors:** Carl C. Anderson, Fabrice G. Renaud

**Affiliations:** 1 School of Interdisciplinary Studies, The University of Glasgow, Dumfries Campus Maxwell House Crichton University, Campus Dumfries, Glasgow, DG1 4UQ UK; 2 School of Interdisciplinary Studies, The University of Glasgow, Dumfries Campus Rutherford/McCowan Building, Crichton University Campus Dumfries, Glasgow, DG1 4ZL UK

**Keywords:** Citizen engagement, Ecosystem-based disaster risk reduction, Framework, Nature-based solutions, Public acceptance, Stakeholder participation

## Abstract

Nature-based solutions (NbS) are increasingly recognized as sustainable approaches to address societal challenges. Disaster risk reduction (DRR) has benefited by moving away from purely ‘grey’ infrastructure measures towards NbS. However, this shift also furthers an increasing trend of reliance on public acceptance to plan, implement and manage DRR measures. In this review, we examine how unique NbS characteristics relate to public acceptance through a comparison with grey measures, and we identify influential acceptance factors related to individuals, society, and DRR measures. Based on the review, we introduce the PA-NbS model that highlights the role of risk perception, trust, competing societal interests, and ecosystem services. Efforts to increase acceptance should focus on providing and promoting awareness of benefits combined with effective communication and collaboration. Further research is required to understand interconnections among identified factors and how they can be leveraged for the success and further uptake of NbS.

**Supplementary Information:**

The online version contains supplementary material available at 10.1007/s13280-021-01502-4.

## Introduction

Public acceptance has become increasingly recognized as a key consideration within natural hazard risk reduction policy (Sarzynski and Cavaliere 2018). At the international level, the Sendai Framework for Disaster Risk Reduction 2015-2030 (UNISDR 2015) codified an “all-of-society” approach that hinges on participation and engagement and includes the words “public” or “society” in seven of its eleven guiding principles. At regional level, perhaps the best example is the European Union Water Framework Directive (European Commission 2000), which requires public participation for addressing flooding in river basin management plans.

In a review of complex environmental risk issues, van der Vegt (2018) argues that a decline in public trust of decision-makers, expert–public disagreements, and greater demand for inclusivity and transparency have motivated the increase in calls for public engagement. Additionally, Wamsler et al. (2019) synthesize motivations for increased citizen involvement in nature-based adaptation planning, citing the burden placed on disaster risk managers in the current context of rapidly changing climatic conditions, citizen–local authority conflicts regarding land-use as a result of these changes, and claims regarding “relevance; fairness; acceptance; and, ultimately, sustainability” (p. 2). Certainly, the push towards increased public engagement can lead to positive outcomes (Reed 2008; Mees et al. 2016). However, gains are predicated on context (Wamsler et al. 2019), and the willingness of the public to accept disaster risk reduction (DRR) efforts and actively engage is not a foregone conclusion (Godschalk et al. 2003).

At the same time, a paradigm shift (back) towards living with, rather than controlling nature (de Groot 2012) has been promoted, spurred by an increasing recognition of synergies among efforts for reducing risk, tackling climate change, and addressing human development issues by leveraging ecosystems and their services (Renaud et al. 2016). With this shift and particularly following the 2004 Indian Ocean Tsunami, ecosystem-based approaches for reducing risks have steadily gained recognition and their uptake continues to grow. These approaches are in contrast to ‘grey’ infrastructure measures such as dykes or seawalls, although the two are often combined in ‘hybrid’ measures.

Various ecosystem-based approaches for reducing risk such as ecosystem-based disaster risk reduction (Eco-DRR) and ecosystem-based adaptation (EbA) or green infrastructure (related to ecosystems on land and/or green spaces) and blue infrastructure (if aquatic ecosystems are involved) now fall under the nature-based solutions (NbS) umbrella concept (Cohen-Shacham et al. 2016). The International Union for Conservation of Nature (IUCN) defines NbS as “Actions to protect, sustainably manage and restore natural or modified ecosystems that address societal challenges effectively and adaptively, simultaneously providing human well-being and biodiversity benefits” (Ibid p. 4). Increasing recognition of the concept is exemplified by the European Commission incorporating NbS as part of its 2020 research agenda and funding a number of large pan-European projects (Faivre et al. 2017). The success of these projects and the continued dissemination of NbS globally will depend on whether the public willingly accepts this approach.

Public acceptance has been a nebulous term as used in literature surrounding sustainability, often employed without a specific working definition (Wüstenhagen et al. 2007). Here, we define the public as a stakeholder group composed of individuals who are affected by the risk reduction measure and reside within or near the measure. Acceptance can be stated or demonstrated and exists on a broad spectrum ranging from rejection to active support (Wüstenhagen et al. 2007). Thus, public acceptance in this context is determined by individual or community attitudes and/or behaviour towards a DRR measure.

The importance of public acceptance varies contextually, but characteristics of NbS suggest that understanding its dimensions and causal determinants is crucial (Cohen-Shacham et al. 2016; Wamsler et al. 2019). The IUCN proposes eight principles that characterize NbS, within which public acceptance is one key theme (paraphrased; 1: embrace nature conservation norms, 2: be implemented alone or in an integrated manner, 3: be determined by site-specific contexts, 4: have fair and equitable benefits with transparency and participation, 5: maintain biological and cultural diversity, 6: be applied at landscape scale, 7: recognize trade-offs between immediate economic benefits and long-term ecosystem services, and 8: be an integral part of the design of methods to address a specific challenge) (Cohen-Shacham et al. 2016, p. 6). For example, the third principle of NbS involves the integration of local and traditional knowledge within site-specific contexts. Knowledge integration is reliant on willing and broad participation, a key theme of the fourth principle. They also suggest that NbS be applied at landscape scale (principle 6) and consider long-term benefits (principle 7). Both principles imply a greater dependence on the public given the inherent value-based trade-offs of land-use and future visions. The multifunctional nature of NbS also creates more potential for value-dependent trade-offs (Nesshöver et al. 2017) as well as the need for multiactor collaborations (Frantzeskaki 2019). This is further supported by several NbS approaches that rely entirely on some degree of public participation, such as Integrated Water Resource Management and Integrated Coastal Zone Management (e.g. Brandolini and Disegna 2015).

More recently, the IUCN has published the Global Standard for NbS. The standard has criteria aligned to the NbS principles but is designed as a more practice-oriented indicator framework for ensuring successful NbS deployment (IUCN 2020). Criterion 5, “NbS are based on inclusive, transparent and empowering governance processes” emphasizes the importance of stakeholder involvement and is the most closely aligned with public acceptance. Criteria 4 and 6 are related to benefits and trade-offs of NbS and also highlight the role of stakeholders for successful NbS deployment (IUCN 2020).

Despite this, past studies on ecosystem-based approaches have focussed primarily on engineering and economic benefits rather than interactions among relevant actors (Triyanti and Chu 2018). Indeed, Kabisch et al. (2016) identify societal relations with NbS specifically as a major knowledge gap, including issues surrounding stakeholder involvement, equity of co-benefits and public communication. One exception is Wamsler et al.’s (2019) assessment of citizen involvement with NbS among Swedish municipalities. Among others, they identify barriers such as a lack of institutional capacity and resources, conflicting public interests, resistance to change, and place attachment. Moreover, they underscore that current organizational structures, often lacking flexibility, may not be conducive to successful citizen engagement, although the advent of NbS offers potential for change. A recent review by Han and Kuhlicke (2019) identifies core topics surrounding perceptions of NbS—co-benefits, risk reduction efficacy, socio-economic and location-specific factors, participation, environmental attitudes, and uncertainty. However, neither study directly compares NbS with grey DRR measures nor considers a set of comprehensive factors that may influence public acceptance and be leveraged to increase it.

These research gaps are reflected in overly generic policy guidelines for societal interactions in the context of NbS approaches. An emphasis is generally placed on stakeholder engagement and participation as instrumental for effectiveness, but recommendations are not tailored for potential unique characteristics of NbS or public acceptance as such. For example, recently published guidelines for design and implementation of ecosystem-based disaster risk reduction and ecosystem-based adaptation by the Convention on Biodiversity (CBD 2019) include a subsection on involving indigenous and local communities (2.3.1) but are largely based on the assumption of public interest and willingness. The following subsection in that document on “mainstreaming” NbS (2.3.2) also exemplifies a lack of systematic consideration of societal interaction within relevant policy guidelines. It emphasizes policy coherence and investment as well as the roles of institutional stakeholders, but disregards public support. However, uptake in policy can also rely on public acceptance, particularly within strong democratic systems.

Determining factors that may contribute to or detract from public acceptance of NbS is crucial given the identified research gaps and increasing investment in NbS projects. Along with providing insight into key areas that merit further research, such factors should allow for guidance towards better design, implementation, and dissemination of NbS. This literature review thus sets out to answer three principal questions:

1. When and why is public acceptance of NbS important and do NbS diverge from grey measures in this regard?
2. What are the factors that influence public acceptance of NbS and do NbS diverge from grey measures in this regard?
3. How can we build public acceptance of NbS by leveraging the identified factors?

Moreover, we integrate the theoretical perspectives of ecosystem services and risk perception of natural hazards to structure key findings. Characterizing NbS benefits from an ecosystem service perspective has been promoted by the IUCN (Cohen-Shacham et al. 2016) and others (e.g. Nesshöver et al. 2017). Risk perception has been used extensively for explaining individual and societal attitudes and behaviours in situations of risk from natural hazards (Terpstra et al. 2006). The results are structured on the basis of these three primary research questions as well as explicit subsections for ecosystem services and risk perception as key concepts. Prior to this, the methods outline the scope of the review and the key word search. Results are followed by a discussion, including limitations of the review and a call for future research guided by a proposed framework for understanding and increasing public acceptance (PA) of NbS—the PA-NbS framework.

## Methods

### Scope

We use three initial scoping criteria for determining which DRR measures are appropriate for the review. Measures must (1) be physical, (2) have public benefits and (3) have natural hazard risk reduction as a primary aim. By limiting the review to blue, green, hybrid, and grey measures, we exclude all measures that do not involve change in the physical environment (e.g. early warning systems). We classify blue, green, and hybrid measures as NbS since they include a natural element and therefore societal co-benefits (Cohen-Shacham et al. 2016). Grey measures are therefore defined by the absence of any natural component.

### Key word search and article screening

We use the Scopus database and ROSES standards for systematic reviews in environmental research (Haddaway et al. 2017). Prior to defining search terms, 11 articles were selected to be included in the review based on expert knowledge and an extensive, non-systematic scan of literature using Scopus and Google Scholar. By ensuring these were found using the key word search, we were able to better train the search process and add confidence to the final composition of search terms.

Based on the guiding research questions for the review, we created three categories of search terms in Scopus applied to titles, key words and abstracts: (1) actors to accept, (2) ways to accept, and (3) DRR and NbS (Table 1). Because the actors listed in Group 1 engage in the actions listed in Group 2, these terms are coupled. For example, articles should include one or more instance of *public acceptance*, *public perception*, *social acceptance*, *social perception*, etc. rather than, e.g. “*public* understanding of cultural *acceptance”*. This was specified in Scopus using the proximity operator “w/2” between the set of group one and group two terms.

**Table 1:** Three search term groups are used and combined with Boolean operators (underlined) to form the search term sequence. All possible pairs of terms from Groups 1 and 2 are created using the operator “w/2”, which connects two words that must be “within two” words each other. An “AND” operator combines these pairs with words from Group 3

Groups		Search terms with unique identifiers
1	Actors to accept (*n* = 10)	(1.1) public, (1.2) social, (1.3) societ*, (1.4) stakeholder, (1.5) communit*, (1.6) individual, (1.7) household, (1.8) resident, (1.9) citizen, (1.10) local
2	Ways to accept (*n* = 17)	(2.1) accept*, (2.2) perception, (2.3) participat*, (2.4) preference, (2.5) buy-in, (2.6) involv*, (2.7) engag*, (2.8) “collective action”, (2.9) sentiment, (2.10) attitude, (2.11) belief, (2.12) behavio*, (2.13) apath*, (2.14) indifferen*, (2.15) burnout, (2.16) fatigue, (2.17) reject*
3	DRR and NbS (*n* = 34)	(3.1) resilien*, (3.2) drr, (3.3) disaster, (3.4) nbs, (3.5) “nature-based solution”, (3.6) “hazard mitigation”, (3.7) “hazard adjustment”, (3.8) “risk mitigation”, (3.9) “risk reduction”, (3.10) “risk management”, (3.11) “risk communication”, (3.12) “eco-engineering”, (3.13) “ecological restoration”, (3.14) “ecological engineering”, (3.15) “forest landscape restoration”, (3.16) “ecosystem-based adaptation”, (3.17) “ecosystem-based mitigation”, (3.18) “climate adaptation services”, (3.19) “ecosystem-based disaster risk reduction”, (3.20) “natural infrastructure”, (3.21) “green infrastructure”, (3.22) “integrated coastal zone management”, (3.23) “integrated water resources management”, (3.24) “protected area management”, (3.25) “ecosystem-based management”, (3.26) “wetland restoration”, (3.27) “floodplain restoration”, (3.28) “building with nature”, (3.29) “natural infrastructure”, (3.30) “river management”, (3.31) “ecosystem services”, (3.32) “landscape restoration”, (3.33) “coastal management”, (3.34) “coastal protection”
Search term sequence^1^		
(1.1 w/2 2.1) OR (1.2 w/2 2.1) OR (1.3 w/2 2.1) OR… (1.1 w/2 2.17) OR (1.2 w/2 2.1) OR (1.2 w/2 2.2) OR (1.2 w/2 2.3) OR… (1.2 w/2 2.17) OR (1.3 w/2 2.1) OR (1.3 w/2 2.2) OR (1.3 w/2 2.3) OR… (1.3 w/2 2.17) OR … (1.10 w/2 2.1) OR (1.10 w/2 2.2) OR (1.10 w/2 2.3) OR… (1.10 w/2 2.17) OR AND 3.1 OR 3.2 OR 3.3 OR… 3.34		

^1^See Supplementary Text S1 for the full search term sequence

To avoid selection bias, we add five key words to Group 2 to capture a potential lack of acceptance (apath*, indifferen*, burnout, fatigue, reject*). For Group 3, we use a list of categories and examples of NbS from a recent IUCN report on NbS (Cohen-Shacham et al. 2016, p. 10). The list is necessary since NbS is still a new term and not always used systematically. The list is not exhaustive, but using the “OR” operator with search terms referring generally to DRR, mitigation, adjustment, and management, we were able to capture relevant physical measures.

We include articles since 1990 and up to May 15, 2019 to be inclusive and since 1990 coincides with an increased awareness of the importance of ecosystems and their societal co-benefits (e.g. the Brundtland Report published in 1987 (Brundtland et al. 1987) and the Rio Earth Summit held in 1992).

**Fig. 1:**
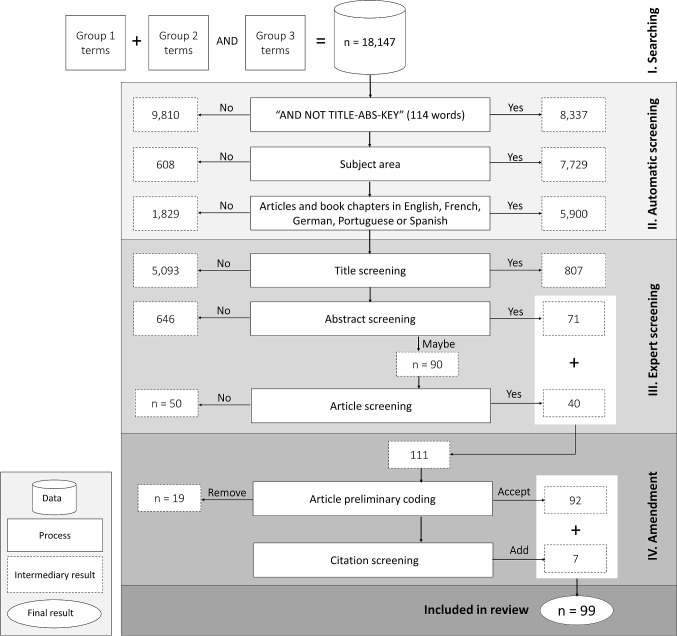
Flow chart of four broad steps (searching, automatic screening, expert screening and amendment) and detailed steps taken to determine the inclusion of articles in the systematic review

All terms in Group 1 and Group 2 were paired, yielding 170 search terms. These terms were connected to Group 3 terms using an “AND” operator, with all terms within groups separated by “OR” operators. The new sequence yielded 18 147 returns in Scopus that were subsequently reduced using a step-wise exclusion methodology (Fig. 1).

We first identified irrelevant terms found in the titles of the first 500 articles (automatically sorted by relevance in Scopus) to exclude thematically divergent articles. We then applied the “filter by subject area” function, only included book chapters and articles in the languages English, French, German, Portuguese or Spanish (being inclusive as possible with language constraints of the reviewers), and removed duplicates.

With the 5 900 articles, we conducted an initial title screening, followed by a screening of abstracts and full articles when necessary. To amend the final 111 article count, all articles were carefully read and 19 more excluded during a round of preliminary coding. This was most commonly due to methodological proposals, bundling behavioural and structural measures in the analysis, or only focussing on technological hazards. All reference sections in the remaining 92 articles were scanned and seven more articles included, resulting in a final total of 99 articles.

### Data extraction

We conducted thematic coding using the software NVivo Pro v.12. In a first reading, all articles were assigned to sets of descriptive categorical classes to better understand the dataset. These identify the case studies described in the articles as either urban/rural, by hazard type, scale, continent, and whether the article describes NbS or grey measures. For the latter, an additional code of “two or more” measure types was created for articles that do not differentiate between NbS and grey measures in their findings. These results are presented in the first results section “Descriptive statistics of the dataset”.

Next, we conducted a round of inductive coding by broadly assigning all explicit or implicit mentions of public acceptance outcomes, influencing factors for acceptance, and ways to increase acceptance to corresponding codes. Subsequent results sections correspond to these three coding exercises. The remaining coding process was inductive and exploratory. Themes were allowed to emerge from the data by starting with this limited set of broad pre-defined codes and iteratively creating new and more detailed categories. These were further disaggregated into more specific themes. In the results section, the findings presented are based entirely on literature from the review dataset. These are the only referenced literature in this section; an exclusive list of which is provided in Supplementary Text S2.

## Results

### Descriptive statistics of the dataset

**Fig. 2:**
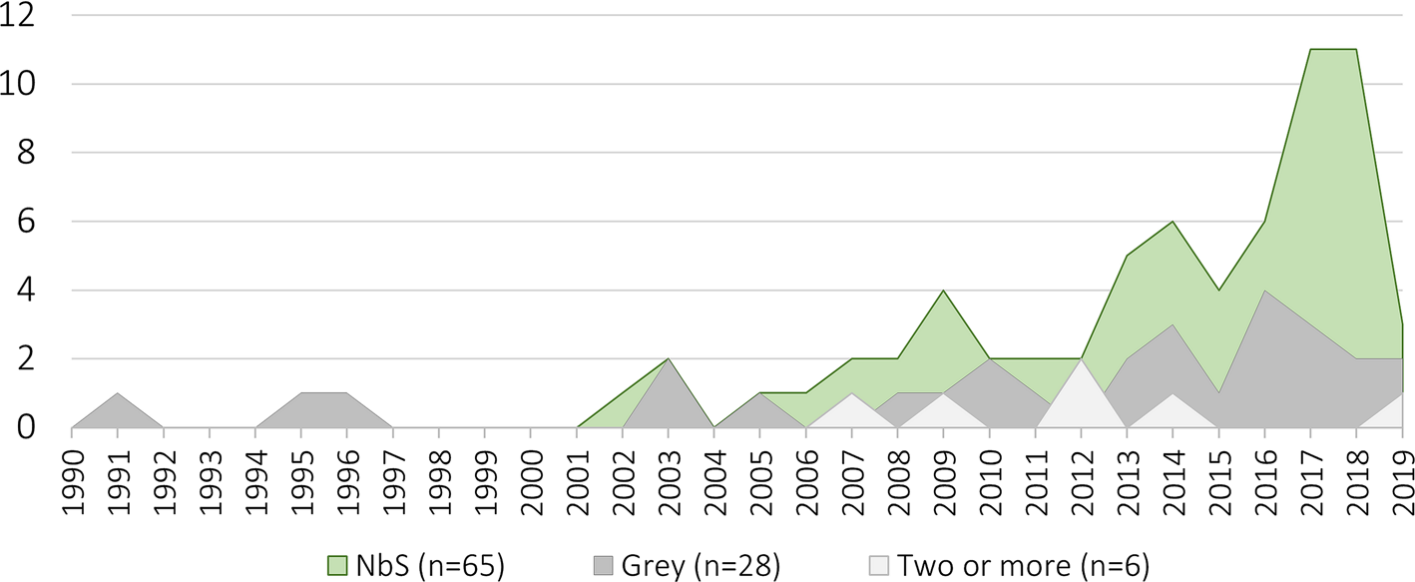
Number of articles describing NbS, grey, or two or more measures by year published included in the literature review

In total, 97 journal articles and two book chapters were selected for coding (a complete list is provided in Text S2), all in the English language except one article in French. A trend of increasing relevant publications since 2001 is evident, particularly for NbS (Fig. 2). Along with an increase in scientific publications generally, this likely reflects both the increase in implementation of participatory approaches and NbS approaches.

Although distinguishing between NbS and grey measures is relatively simple, grouping measures based on their underlying concepts is more difficult. This is a result of the breadth and complexity of terms used as well as their overlap. Relying primarily on how the authors define their own work, the most common forms of NbS in the review are ecological restoration (*n* = 17), risk and ecosystem management (*n* = 15), green and blue-green infrastructure (*n* = 13), and managed realignment (*n* = 6) (Table S1). Only one article makes explicit reference to NbS. For grey measures, descriptions are more generic due in part to less terminological/conceptual competition, the most common being simply “structural measures” (*n* = 6).

**Fig. 3:**
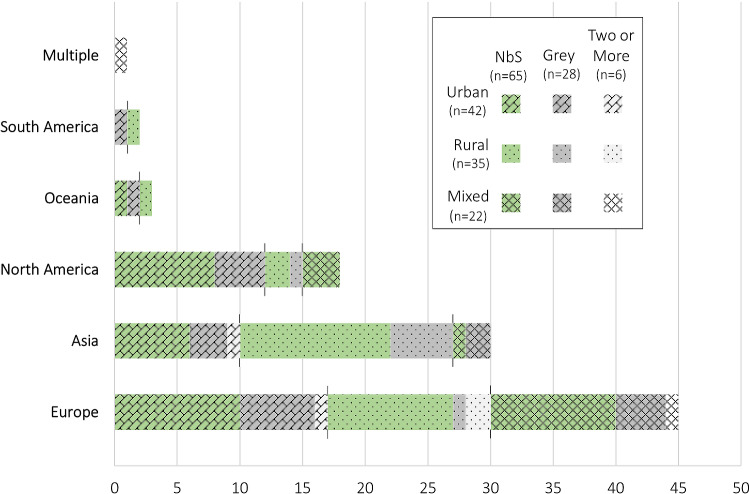
Number of articles describing NbS, grey, or two or more measures in urban, rural, and mixed contexts by continent included in the literature review. No reviewed articles describe measures in Africa

The most common type of article describes rural NbS in Asia (*n* = 12), driven by mangrove replanting/restoration. The second most common article type is NbS in an urban (*n* = 10), rural (*n* = 10) or mixed (*n* = 10) context in Europe (Fig. 3). There is considerable variation in the dataset, although there are no studies from Africa and only five between South America and Oceania.

**Fig. 4:**
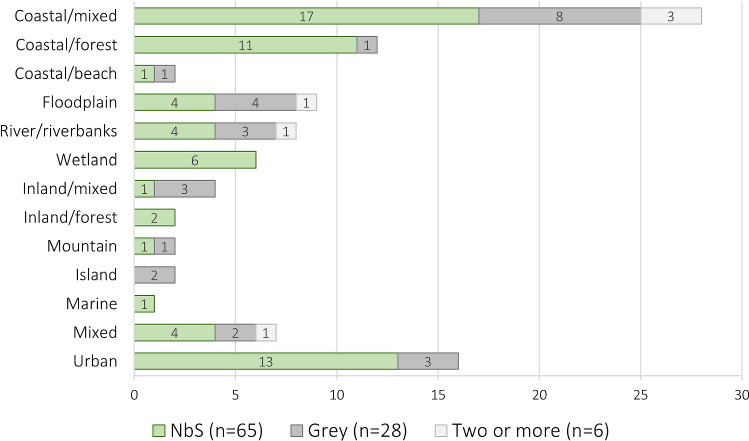
Number of articles describing NbS, grey, or two or more measures by land cover included in the literature review. “Mixed” denotes multiple land covers across geographies, while “Coastal/mixed” and “Inland/mixed” denote mixed land-use within these respective geographies

Nearly half of all articles describe measures implemented in a coastal setting (*n* = 42). Despite some variation in land covers, low-lying areas are greatly overrepresented in the dataset, including also floodplains (*n* = 9), (low-lying) rivers (*n* = 8) and wetlands (*n* = 6). Comparing NbS to grey measures in these environments, the influence of mangrove restoration as a coastal forest NbS and ecological restoration of wetlands is pronounced (Fig. 4).

Only 16 articles are classified as urban land cover because measures focussing on rivers or riverbanks, for example, may occur within cities but are classified at this more specific level. Measures with urban land cover most often involve urban storm water, such as “sponge city” or SuDS (sustainable urban drainage system) designs.

**Fig. 5:**
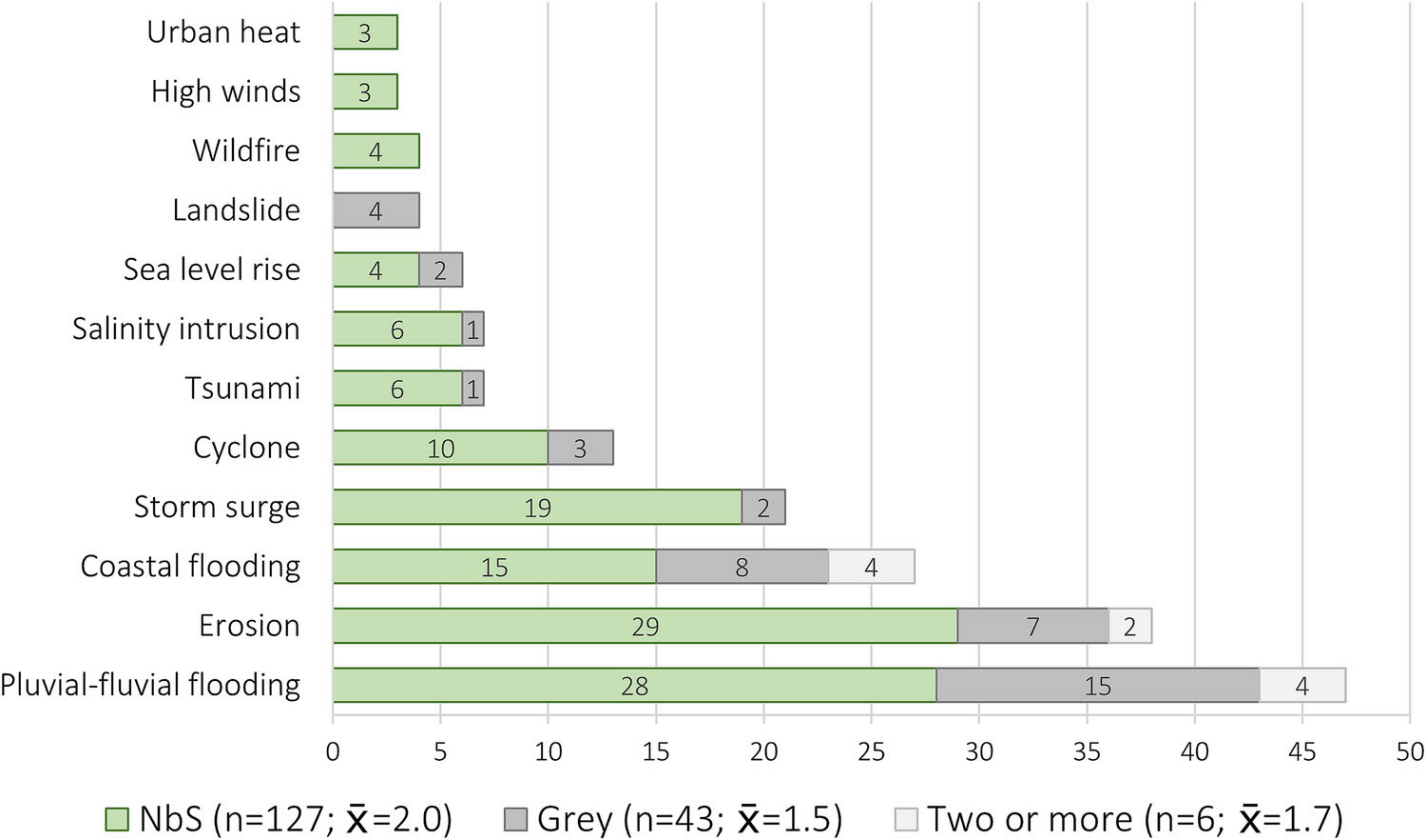
Number of articles describing NbS, grey, or two or more measures aimed at reducing risk from different natural hazards included in the literature review. The total number of hazards addressed by each measure type and corresponding arithmetic mean are provided

Twelve different hazards were identified in the articles, with flooding being the most prominent (Fig. 5). Many measures, particularly NbS, address multiple hazards (on average, two hazards per NbS article and 1.5 hazards per grey article). This is driven in part by the stated aim of coastal NbS to reduce erosion as a secondary benefit along with more sudden-onset coastal hazards like storm surge.

### When and why is public acceptance of NbS and grey measures important?

There are many positive, negative, and neutral indicators and manifestations of acceptance in the reviewed literature (Table S2). As a consequence of these manifestations and indicators, we identify twelve broad benefits of public acceptance for DRR measures relevant to specific project phases (Table 2). For example, public provision of labour can reduce the cost of the measure (Abbas et al. 2016). This form of acceptance is most often referenced regarding the maintenance and management project phase (e.g. Barbier 2006), although cooperative implementation (e.g. Triyanti et al. 2017) and cooperative monitoring and evaluation (e.g. Verbrugge et al. 2017) are also cited. Public acceptance in relation to these latter two project phases is mentioned more in the context of NbS than grey measures. Examples include relying on local villagers to provide labour for mangrove replanting in Thailand (Barbier 2006) and Indonesia (Triyanti et al. 2017), and working with landowners in the context of managed realignment in the U.K. (Esteves and Thomas 2014) and fire management in Australia (Ryan and Wamsley 2008). The landscape scale and long-term nature of these measures, their reliance on limited and/or bottom-up funding, as well as their embeddedness within social–ecological systems increases reliance on public acceptance. Moreover, the relevance of monitoring and evaluation of such NbS is crucial given their long time-lines and lag-times between implementation and benefits (Verbrugge et al. 2017). Although ‘cooperative maintenance and management’ is not distinguished as a much more common benefit among articles that describe NbS compared to grey measures, ‘sustainable use’ is. This can be considered a form of maintenance, since overexploitation of (e.g. mangrove) resources could lead to degradation and ineffectiveness of the measure itself (Barbier 2006).

**Table 2:**
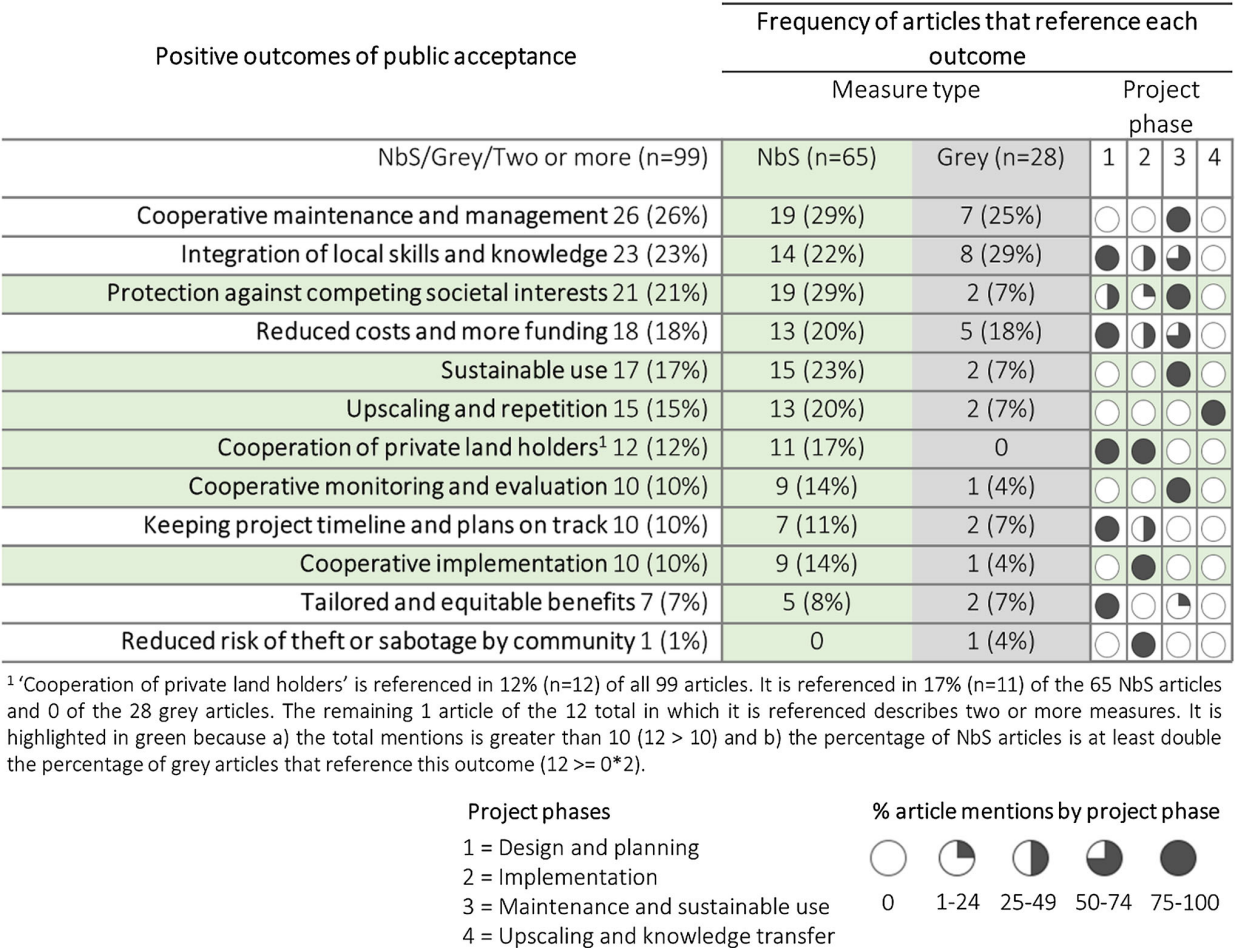
Positive outcomes of public acceptance by measure type and project phase listed from highest frequency to lowest frequency considering all the articles (*n* = 99; including articles describing NbS [*n* = 65], grey measures [*n* = 28], and two or more measures [*n* = 6]). The second column *(green)* shows the number and percentage of NbS articles (out of the 65 total) that reference each outcome in relation to public acceptance. The third column (*grey)* replicates this for articles describing grey measures. An outcome’s row is highlighted in green if the outcome a) occurs in *n* ≥ 10 total articles and b) the percentage of NbS articles that reference it is at least double the percentage of grey articles that reference it. An example is provided in the footnote of the table

There are higher percentages of NbS articles that describe positive outcomes of public acceptance for NbS compared to articles describing grey measures. This suggests that public acceptance is generally more important for the success of NbS when compared to the success of grey measures. Moreover, there are a number of positive outcomes that are much more relevant to NbS than grey measures, but not vice versa (based on the percentages in Table 2). For example, the outcome of ‘sustainable use’ illustrates the embeddedness of NbS in society, which also makes them particularly susceptible to changes in land-use and competing societal interests, both in the short- and long-term. Holstead et al. (2017) and Schaich (2009) describe natural flood management as conflicting with agricultural food production and therefore susceptible to farmers’ perceptions. Moreover, Everett et al. (2018) describe blue-green infrastructure as more likely to be an object of public perceptions and attitudes than grey infrastructure since blue-green infrastructure often more drastically alters the landscape.

Acceptance leading to upscaling and repetition is also highlighted as being more relevant for NbS than grey measures. The novelty of NbS and associated lack of confidence in their effectiveness may make their dissemination more difficult (Buchecker et al. 2015; Chou 2016), although their aesthetic and pro-environmental appeal is promising in this regard (Buijs 2009).

Public acceptance is shown to be important throughout project phases. However, there is some indication of increased importance in the design and planning phase (phase 1) and again during maintenance and sustainable use (phase 3) (Table 2). The former likely reflects a threshold during the planning stage for preventing outright public rejection (Godschalk et al. 2003; Davis and Cole 2004). The phase of ‘maintenance and sustainable use’ is also related to the embeddedness of the measures, particularly NbS, within social–ecological systems. Although upscaling and knowledge transfer was rarely explicitly connected to other outcomes of public acceptance, it should be seen as feeding back into the design and planning phase.

### What factors influence public acceptance of NbS and grey measures?

In total, we identify 36 interconnected factors that influence public acceptance of NbS and grey measures (Table S3). Here, factors referenced in at least five different articles are listed in order of frequency, although their importance for public acceptance is highly contextual (Table 3). We group the factors based on their characterization of the measure (and project, when relevant), the individual, or the society. Some societal factors are often attributed to individuals in the articles, but are classed as such because of their social nature (e.g. place attachment, trust). Although many of these factors are shared for NbS and grey measures, there are clear distinctions in their importance for each measure type as evidenced by their prevalence within the respective reviewed literature. In particular, the benefits and trade-offs of the measures, their perceived effectiveness, relevant costs and funding, an awareness and understanding of the measure, a sense of responsibility for the measures, public participation, and competing societal interests all emerge as more relevant for NbS than grey measures and are highlighted in the table below.

**Table 3:**
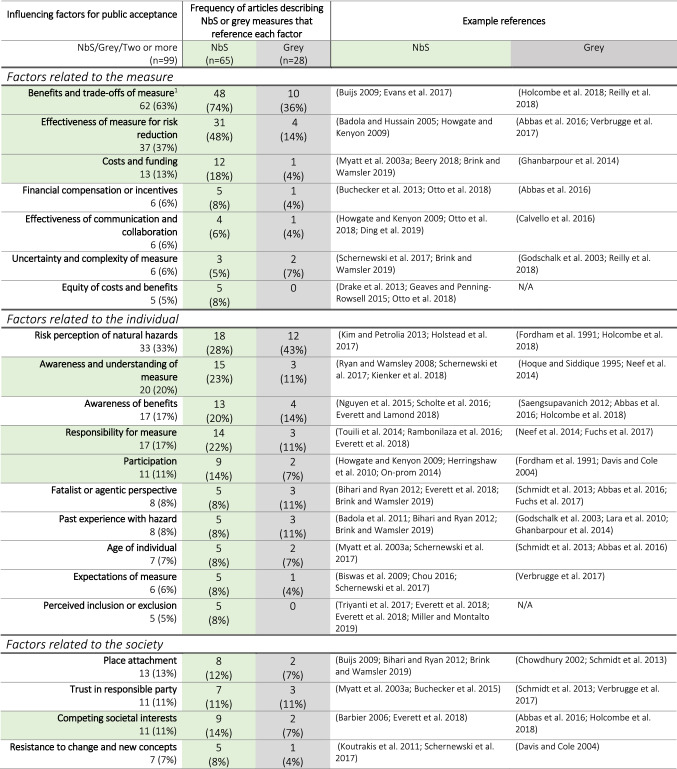
Influencing factors for public acceptance grouped by relation to the measure, the individual, or the society. Within these groupings, the factors are listed from highest frequency to lowest frequency considering all the articles (*n* = 99; including articles describing NbS [*n* = 65], grey measures [*n* = 28], and two or more measures [*n* = 6]). The second column *(green)* shows the number and percentage of NbS articles (out of the 65 total) that reference each factor in relation to public acceptance. The third column (*grey)* replicates this for articles describing grey measures. A factor’s row is highlighted in green if the factor (a) occurs in *n* ≥ 10 total articles and (b) the percentage of NbS articles that reference it is at least double the percentage of grey articles that reference it. An example is provided in the footnote of the table

^1^Benefits and trade-offs of measure’ is referenced in 63% (*n* = 62) of all 99 articles. It is referenced in 74% (*n* = 48) of the 65 NbS articles and 36% (*n* = 10) of the 28 grey articles. The remaining four articles of the 62 total in which it is referenced describe two or more measures. It is highlighted in green because a) the total mentions is greater than 10 (62 > 10) and b) the percentage of NbS articles is at least double the percentage of grey articles that reference this factor (74 ≥ 36*2)

### Factors related to the measure

Benefits and trade-offs are the most frequently mentioned among all the factors that influence public acceptance. ‘Benefits’ includes both the perceived primary function of the measure as well as any co-benefits. The frequencies for NbS and grey measures suggest more importance of a broader range of benefits for NbS. Given their importance for NbS, we use the concept of ecosystem services to further explore which specific benefits are most relevant in the following subsection.

The effectiveness of the measures for risk reduction is also a primary public concern—an unsurprising finding given that this is a principal goal of the measures in the reviewed articles. In 21 of the 37 articles that mention this factor, scepticism about the measure reduces acceptance. Of these, 18 describe NbS. A lack of evidence (Esteves and Thomas 2014), a belief in the displacement rather than reduction of risk (Davenport et al. 2010) and a greater trust in alternative grey measures (Chou 2016) help explain this tendency. Another factor, the uncertainty and complexity of the measure, is closely related since it can make awareness and understanding of NbS more difficult (Schernewski et al. 2017). Confidence in effectiveness for both measure types was often a result of past experiential evidence, gained through project participation (Buchecker et al. 2013), regular exposure to the measure (Kim and Petrolia 2013), or merely observation over time (Ding et al. 2019). The duration of implementation and time-lag for effectiveness of NbS is related to complexity and creates a broader time window for public dissent (Schernewski et al. 2017).

We also identify costs and funding as a crucial factor, mentioned in reference to NbS with only the exception of Ghanbarpour et al. (2014). In terms of influencing acceptance, cost is inextricably linked to perceived value (Everett et al. 2018), which in turn is also associated with perceived effectiveness of the measure.

#### Provision of ecosystem services

**Fig. 6:**
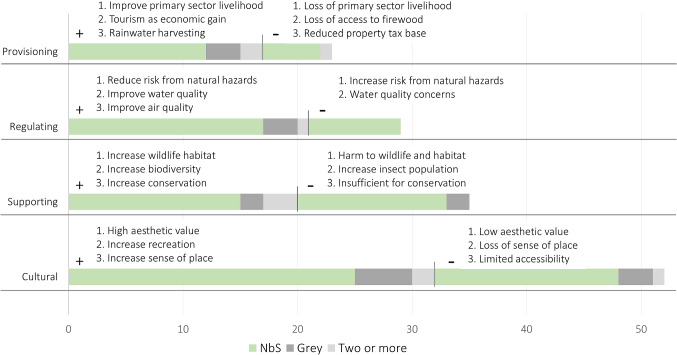
Number of articles in the review describing NbS, grey measures, or two or more measures that associate public perception of each ecosystem service (cultural, supporting, regulating, provisioning) with public acceptance of the measures. For each ecosystem service, there are positive associations (“+” i.e. lead to increased acceptance) and negative associations (“−”i.e. lead to decreased acceptance). The three specific ecosystem services mentioned the most by the three article types are shown for each ecosystem service category and direction

Since 59 of the 62 articles mentioning benefits draw an implicit or explicit connection between ecosystem services and acceptance, we describe the co-benefits of the measures using the Millennium Ecosystem Assessment typology for ecosystem services (MEA 2005), (Figs. S1; S2). Descriptions of an increase in acceptance are found in 48 articles, while descriptions of a decrease in acceptance are found in 30 articles. Several other articles (also) include descriptions of neutral or insignificant connections (*n* = 8). Although most of these articles describe NbS (*n* = 47), case studies of grey measures (*n* = 8) also include a link between ecosystem services and public acceptance. Examples of the latter case include concrete drains that reduce landslide risk as well as stagnant water that can breed mosquitos (Holcombe et al. 2018), and a dam providing recreation opportunities (Reilly et al. 2018). For both NbS and grey measures, cultural services are the most prevalent in relation to acceptance. Within this category, high or low aesthetic value is mentioned the most as either increasing or decreasing acceptance, respectively (Fig. 6). Other predominant cultural services include recreation opportunities arising from ecological restoration (e.g. Kim and Petrolia 2013) and either preservation of sense of place (e.g. Buijs 2009) or loss of sense of place through change (e.g. Goeldner-Gianella et al. 2015).

We include in supporting services general descriptions of benefits such as changes in habitat, biodiversity and conservation, since these contribute to other service types. Of the negative associations for each category, those describing supporting services form the largest percentage within any category. This is driven by perceived or anticipated harm to wildlife and habitat [*n* = 8] (e.g. Koutrakis et al. 2011) and increased number of insects due to habitat provision, including mosquitos [*n* = 5] (e.g. Scholte et al. 2016). Global climate regulation is only mentioned in three of the articles, two in a positive context (Brink and Wamsler 2019; Miller and Montalto 2019) and one in which it is seen as suppressing altruistic motivations for acceptance given its widespread rather than local provision (Drake et al. 2013).

The most common regulating service is the ability of the NbS to reduce risk from the relevant natural hazards. Coastal hazards (*n* = 11) and pluvial/fluvial flooding (*n* = 6) are the two most common hazards in this category (see Fig. S1 for the detailed composition of ecosystem services and disservices). Several articles also mention an improvement in quality of air (Miller and Montalto 2019) and water (Holcombe et al. 2018) as regulating services. Nearly half of the articles mentioning provisioning services describe mangrove planting or conservation efforts and refer most often to protection or enhancement of primary sector livelihoods (*n* = 11) related to fishing (e.g. Evans et al. 2017) or agriculture (e.g. Badola and Hussain 2005).

### Factors related to the individual

The degree of perceived risk of natural hazards by individuals as a factor for determining acceptance is mentioned in 33 articles, more than any other factor related to the individual. Given the frequency, complexity and highly contextual nature of this factor, we devote a separate subsection to it below.

‘Awareness and understanding of the measure’ is also crucial to acceptance, even more so for NbS than for grey measures. For example, Kienker et al. (2018) found that more informed residents were willing to pay more for ecological engineering in Australian harbours. For managed realignment schemes in the U.K., Myatt et al. (2003a) and Myatt-Bell et al. (2002) show that residents who consider themselves aware and well-informed are more convinced by their efficacy. Likewise, misconceptions of NbS, including misaligned expectations caused by overly technical language (Chou 2016), past financial incentives (Biswas et al. 2009), or high public standards for safety (Geaves and Penning-Rowsell 2015) can have antagonistic effects. Complexity and novelty of NbS also exacerbate this compared to grey measures (e.g. van den Hoek et al. 2014; Schernewski et al. 2017).

Closely connected to an understanding of the measure is an understanding of its benefits, found to be important for both NbS and grey measures. A low awareness of benefits was cited as reducing acceptance (*n* = 9) more often than a high awareness increasing acceptance (*n* = 6). For the former, focussing on a limited number of specialized benefits (Davenport et al. 2010), inadequate monitoring and reporting of benefits (Nguyen et al. 2015), and misattribution of benefits [i.e. to something other than the measure] (Everett and Lamond 2018) were highlighted as causal factors. Appreciation of more hidden ecosystem service benefits like climate change mitigation, wildlife corridors (Everett et al. 2018) and habitat provision (Badola et al. 2011) is often lacking.

A sense of responsibility for the measure can also act to increase or decrease acceptance. Nine articles reference a displacement of responsibility from individuals to e.g. the state (e.g. Buchecker et al. 2015), resulting in disinterest or unwillingness to participate or collaborate. A sense of burden of responsibility was described in seven of the articles, in which a feeling of liability for maintenance was prevalent (e.g. Everett et al. 2018). This is more of an issue for NbS than grey measures given their greater reliance on maintenance by the public. A positive sense of responsibility can also lead to ownership, described as being fostered by social altruism (Brink and Wamsler 2019) or project participation (On-prom 2014). Project participation is not only a potential indicator of acceptance, it is also identified as leading to trust and knowledge exchange (Herringshaw et al. 2010), spreading awareness (On-prom 2014), and aligning expectations of the measure (van den Hoek et al. 2014), all potentially feeding back into public acceptance.

#### Risk perception

Nearly all of the 33 articles that link risk perception of natural hazards to acceptance do not disaggregate the concept of risk but rather assess it as a general idea and often refer to related concepts such as perceived concern (Ding et al. 2019), consequences (Bubeck et al. 2012), fear (Rambonilaza et al. 2016), or threat (Schaich 2009).

Generally, a higher perceived risk of the hazards is described as leading to more acceptance of both NbS and grey measures (e.g. Chowdhury 2002; Rambonilaza et al. 2016; Everett et al. 2018). However, several articles also consider risk perception but find no significant directional relation with acceptance. de Groot and de Groot (2009) and Schernewski et al. (2017) equate this to the lack of substantial “objective” flood risk within the Netherlands and Germany, respectively. In the cases of Schaich (2009) and Kim and Petrolia (2013), the co-benefits of ecological restoration for flood risk reduction increase public support regardless of risk perception. This illustrates that the co-benefits of NbS can have more influence on acceptance than perceived risk and risk reduction capacity of measures. However, Kim and Petrolia (2013) also find that support for wetland restoration in the Mississippi Delta declines among respondents who perceive a high frequency of category 3 hurricanes or greater. Likewise, Goeldner-Gianella et al. (2015) make a connection between fear and acceptance of depolderization. They suggest that a lack of fear of coastal storms in the U.K. has led to relatively greater acceptance of depolderization, whereas higher risk perceptions due to past hazard events in France and Germany have had the opposite effect.^1^ This suggests that once a certain threshold of perceived risk has been met, the perceived effectiveness of the measure strongly modulates acceptance.

Along with risk perception and effectiveness, people’s acceptance of risk or risk (in)tolerance also seems to be an important explanatory factor. Buchecker et al. (2015) describe a low tolerance for damages from natural hazards among residents in the Swiss Alps increasing the demand for risk reduction measures from the state. Chowdhury (2002) assesses residents’ “preparedness to live with flooding” and finds an association with the perceived importance of embankments in Dhaka, while Holstead et al. (2017) find that if farmers are not “bothered by flooding” they are less likely to implement natural flood management plans.

The literature suggests three key differences between NbS and grey measures regarding the relation between risk perception and acceptance. Co-benefits of NbS can foster acceptance in the absence of high risk perception (Schaich 2009), while the complexity, novelty, and lack of evidence for the effectiveness of NbS can negate support in contexts of higher perceived risk (Goeldner-Gianella et al. 2015). Lastly, the “lulling effect” (a false sense of security due to exaggerated perceived effectiveness of the measure), was cited as influencing risk perception only due to grey measures (e.g. Kuo et al. 2015) but not NbS.

**Fig. 7:**
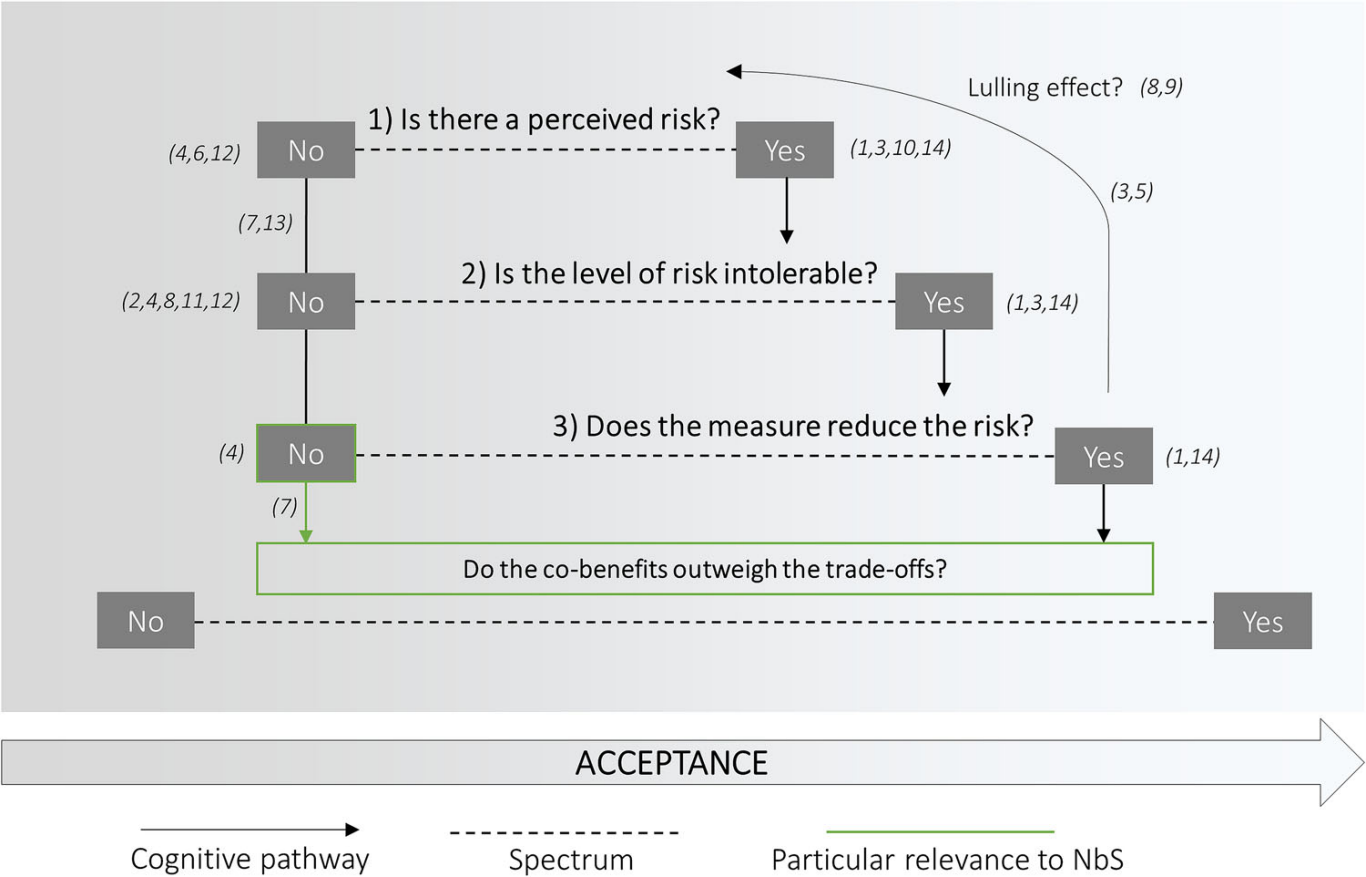
The “Risk Perception—Measure Acceptance Model” or RP-MAM is presented as a decision tree with three ordered questions—(1) Is there a perceived risk, (2) Is the level of risk intolerable, and (3) Does the measure reduce the risk? In this way, risk perception is modulated by risk tolerance and the latter modulated by perceived effectiveness. The respective answers fall on a spectrum that suggests either more or less acceptance of the measure. The final question also feeds back into the perceived risk, potentially creating a lulling effect of low risk perception. Co-benefits of measures, particularly of nature-based solutions, are included as possibly modulating acceptance more than the three risk-related questions, given that risk reduction is often not the primary perceived benefit. References in the figure match the phenomenon in the model to observations in the corresponding articles. Note that multiple observations are possible in the same article. (1) Badola et al. (2011); (2) Brink and Wamsler (2019); (3) Bubeck et al. (2012); (4) Chowdhury (2002); (5) de Groot and de Groot (2009); (6) Fuchs et al. (2017); (7) Goeldner-Gianella et al. (2015); (8) Holstead et al. (2017); (9) Kuo et al. (2015); (10) Myatt et al. (2003a, b); (11) Neef et al. (2014); (12) Rambonilaza et al. (2016); (13) Schaich (2009); (14) Schmidt et al. (2013)

Clearly, the link between risk perception and acceptance of the risk reduction measures is often more complex than a linear relation and involves other mediating factors. Risk perception, mentioned in 33% of all articles, is the third most commonly mentioned factor that can influence acceptance of NbS in the reviewed literature (Table 3) and related to the two most commonly mentioned factors—perceived benefits and trade-offs (63%) and perceived effectiveness for risk reduction (37%). Given their importance and interconnections, we present a generalized theoretical model to link these concepts. The “Risk Perception—Measure Acceptance Model” or RP-MAM takes the form of a decision tree that depicts the relation between these factors (Fig. 7).

### Factors related to the society

Place attachment is referenced in 13 articles as a factor for increasing or decreasing acceptance, more than any other societal factor. Support is shown for both NbS and grey measures that help preserve place (Chowdhury 2002; de Groot and de Groot 2009; Bihari and Ryan 2012), while strong opposition is shown to measures that shift from the status quo or the idealized environment (Roca and Villares 2012). In the context of NbS, Goeldner-Gianella et al. (2015) and Pueyo-Ros et al. (2018) describe a high degree of local attachment to coastal promenades under threat from depolderization and wetland restoration, respectively. Measures are opposed among residents with higher place attachment due to changes in place and services, despite the fact that a wilder coastline would provide overall greater benefits to a broader swath of society. Similarly, Buijs (2009) finds that residents in the Netherlands feel less attached to floodplains after restoration, since local narratives, personal memories and a sense of what is “typical Dutch” are degraded.

High levels of trust and high acceptance are associated in three articles, while low trust reducing acceptance is more prominent (*n* = 8). For both NbS and grey measures, trust was eroded by a fear of hidden agendas (Davenport et al. 2010), insufficient long-term investment (Myatt et al. 2003a), past failed or inadequate measures (Davis and Cole 2004; Schmidt et al. 2013), and low perceived technical competence for implementation (Ryan and Wamsley 2008). Past positive experiences of dealing with flooding in Switzerland (Buchecker et al. 2015) and the Netherlands (Verbrugge et al. 2017) and interacting with green infrastructure in China (Ding et al. 2019) increased public trust in authorities.

The factor ‘competing societal interests’ was found to be much more relevant for NbS than grey measures. Barbier (2006), Davenport et al. (2010), and Badola et al. (2011) indicate that more immediate quality of life concerns related to poverty can take precedent over support for ecological preservation or restoration. Both open green/blue spaces for flood risk management in the U.K. and bioswales in the U.S. met resistance due to perceived impact of decreased parking space and increased traffic (Everett et al. 2018; Everett and Lamond 2018).

### How to increase public acceptance of NbS?

We categorize the coded interconnected ways to increase public acceptance of NbS suggested in the literature into four overarching non-chronological recommendations: provide benefits, increase awareness of benefits, communicate effectively, and promote participation and collaboration (Table 4). These broad categories, as well as the brief explanatory statements below them, represent our own classification of the coded segments. These are further broken down into four corresponding principal success criteria each, derived from sub-themes that emerge from the coded segments. We do not include a measure of confidence in the recommendations but rather aim to create a comprehensive “library” of all recommendations derived from the reviewed literature. The importance of each recommendation is context dependent. Although many of the same recommendations hold true for grey measures, we base these criteria on articles describing NbS and aim to highlight its aforementioned distinguishing characteristics.

**Table 4:** Recommendations for increasing public acceptance of NbS are categorized by four general considerations: provide benefits, increase awareness of benefits, communicate effectively, and promote participation and collaboration, which are further disaggregated into four corresponding success criteria

Success criteria	Recommendations/examples	Example citations
*Provide benefits*		
**Multifunctional** Co-benefits and broad but definable goals are crucial to acceptance	Improve aesthetics	Buijs (2009), Chen et al. (2018)
	Restore cultural elements	Davenport et al. (2010)
	Create synergies with community economic goals	Davenport et al. (2010)
	Support livelihoods	Badola and Hussain (2005), Biswas et al. (2009)
**Equitable** Benefits are subjective and can accrue differently in time and space, creating inequity	Ensure effective communication and participation in decision-making	Roca and Villares (2012)
	Create a common vision and equitable outcomes	Schmidt et al. (2013)
	Redistribute benefits	Drake et al. (2013)
	Improve livelihoods	Badola and Hussain (2005), Biswas et al. (2009)
**Tangible** When benefits to residents are tangible, their impact is felt rather than passively acknowledged	Provide physical benefits (e.g. creating a bike or canoe rental as a part of wetlands restoration project)	Davenport et al. (2010)
	Make benefits as immediate as possible for attribution and early acceptance	Biswas et al. (2009)
	Prioritize subtle and effective changes rather than major overhauls	de Groot and de Groot (2009)
**Non-competitive** Although all NbS involve change and inevitable trade-offs, these should be limited and/or compensated when possible	Implement landscape measures on, e.g. less productive agricultural land	Holstead et al. (2017)
	Find synergies with prominent community issues like transportation, zoning, or development	Godschalk et al. (2003)
*Increase awareness of benefits*		
**Attributable to the measure** The more people recognize what the NbS is providing them, the more likely they are to be supportive (Trialfhianty and Suadi 2017)	Consider the full range and spatial scope of benefits in information and education campaigns	Davenport et al. (2010), Brandolini and Disegna (2015), Everett et al. (2018), Miller and Montalto (2019)
	Use ecosystem services as a theoretical starting point for identifying and conveying benefits for public understanding	Chen et al. (2018)
	Inform about what the NbS cannot provide, including the trade-offs of the measure, so that misaligned expectations are avoided	Kuo et al. (2015), Miller and Montalto (2019).
**Salient** Public recognition of “hidden” benefits is key. *How* risk is reduced may be hidden—e.g. the capacity of wetlands to regulate flooding (Davenport et al. 2010) or urban green infrastructure for heat (Miller and Montalto 2019) or flood reduction (Chou 2016)	Increase visibility of benefits by improving access to NbS areas	Schernewski et al. (2017), Miller and Montalto (2019)
	Demonstrate benefits through public participation (e.g. monitoring or citizen science)	Holstead et al. (2017)
	Emphasize hidden co-benefits if these are of value (e.g. conservation, water purification, or soil formation)	Davenport et al. (2010), Geaves and Penning-Rowsell (2015), Chen et al. (2018), Pueyo-Ros et al. (2018)
**Evidence-based** The novelty and complexity of NbS can breed scepticism, making proof of effectiveness critical	Clearly communicate quantifiable costs and benefits to increase transparency and trust while also aligning public expectations	Esteves and Thomas (2014), Goeldner-Gianella et al. (2015), Holstead et al. (2017)
	Use other comparable and successful sites as proofs of concept	Roca and Villares (2012)
	Conduct experiments and long-term monitoring to provide evidence on-site after implementation	Evans et al. (2017)
**Culturally significant** Benefits are only meaningful in contexts of values. Thus, they should be value-framed based on what is perceived as important or prevailing social norms (Everett and Lamond 2018)	Appeal to safety interests	Everett and Lamond 2018)
	Appeal to economic/livelihood interests	Bubeck et al. (2012), Goeldner-Gianella et al. (2015), Everett and Lamond (2018)
	Appeal to environmental or biodiversity interests	Ryan and Wamsley (2008), Everett and Lamond (2018)
	Appeal to aesthetic interests	Schmidt et al. (2013), Chen et al. (2018), Miller and Montalto (2019)
	Appeal to educational interests	Schmidt et al. (2013), Chen et al. (2018), Miller and Montalto (2019)
	Appeal to place (e.g. sense of community) interests	Schmidt et al. (2013), Chen et al. (2018)
	Appeal to people’s sense of self-efficacy	Everett and Lamond (2018)
*Communicate effectively*		
**Clear and consistent** Communication should foster understanding and knowledge transfer	Make communication strategies anticipatory and adaptive	Davis and Cole (2004), Schernewski et al. (2017)
	Design communication strategies to increase awareness of the measure and justify the rationale behind the measure (e.g. why here?, why now?)	Esteves and Thomas (2014), Schernewski et al. (2017)
	Maintain close and regular contact with the media and prepare outreach materials and articles	Schernewski et al. (2017)
	Stay on message	Esteves and Thomas (2014)
	Open communication channels already in the planning stage and sustain them	Kuo et al. (2015), Schernewski et al. (2017)
	Use plain language, particularly for risk communication	Davenport et al. (2010), Kuo et al. (2015), Chou (2016)
	Include relevant time-frames and targets so people know what to expect and when	Myatt et al. (2003a), Esteves and Thomas (2014), Everett et al. (2018)
**Two-way and multipath** Communication both to and from project managers fosters learning, but only through accessible channels	Create opportunities for communication that are active and dialogic	Howgate and Kenyon (2009), Everett et al. (2018), Everett and Lamond (2018)
	Establish trust, common understanding, and social capital through collaborative and goal-oriented dialogues	Biswas et al. (2009), Howgate and Kenyon (2009), Calvello et al. (2016), Triyanti et al. (2017)
	Facilitate sustained access to two-way dialogue	Kuo et al. (2015), Holstead et al. (2017)
	Make use of formal and informal communication pathways, since highly structured formats can limit involvement	Davenport et al. (2010), Scholte et al. (2016)
	Use a wide range of communication channels (e.g. internet, social media, radio, newspaper)	Howgate and Kenyon (2009), Howgate and Kenyon (2009), Chou (2016), Schernewski et al. (2017), Chen et al. (2018)
	Use trusted and established networks for information dissemination	Bihari and Ryan (2012), Calvello et al. (2016)
**Value-framed** Communication (not just to increase awareness of benefits) can be framed in a way that appeals to the public and follows important (contextually dependent) public narratives	Emphasize mutual attachment to community and place, fostering a sense of altruism and shared responsibility	Bihari and Ryan (2012), Holstead et al. (2017), Beery (2018), Brink and Wamsler (2019)
	Appeal to environmentally conscious citizens with environmental information	Buchecker et al. (2015), Chou (2016), Beery (2018)
	Highlight quality of life concerns if these are preeminent, as is often the case	Godschalk et al. (2003), Chou (2016), Miller and Montalto (2019)
	Make use of targeted messaging when possible, since the ‘public’ is not a homogenous entity	Myatt et al. (2003a)
**Place-based** Communication should be grounded with local relevance	Provide information at the most understandable and relevant scale possible	Myatt et al. (2003a)
	Describe how spatial scales interact (e.g. how the measure fits into a larger context)	Holstead et al. (2017)
	Link outreach to existing community groups and established networks	Davenport et al. (2010), Tanaka et al. (2011), Bihari and Ryan (2012), Triyanti et al. (2017)
	Make use of testimonies from in-groups and locally trusted intermediaries	Bihari and Ryan (2012), Holstead et al. (2017)
	Explain any short- and long-term changes and impacts to place	de Groot and de Groot (2009), Davenport et al. (2010), Kienker et al. (2018)
	Describe the history of hazard events as a reminder and a justification for the measure	Godschalk et al. (2003), Chou (2016)
	Be sensitive to and consider integrating local causal explanations (e.g. for hazard events)	Neef et al. (2014)
	Consider local subjective risk tolerance rather than assuming risk to be a motivating factor	Calvello et al. (2016)
*Promote participation and collaboration*		
**Early and sustained** Efforts should be based on public input and foster a sense of both self-determination and trust with project managers	Involve citizens already in the design and planning phase (e.g. co-determine goals and indicators)	Davis and Cole (2004), Davenport et al. (2010), Schmidt et al. (2013)
	Devote resources to gaining early acceptance by, e.g. integrating local knowledge, which can also increase measure effectiveness	Pueyo-Ros et al. (2018)
	Demonstrate commitment to long-term benefits with sustained public-project manager interactions	Davenport et al. (2010), Herringshaw et al. (2010), On-prom (2014)
**Broad and inclusive** Members of the public are diverse and have different skills and capabilities	Craft many different opportunities and options for the public to get involved and to volunteer	Davenport et al. (2010), Chou (2016), Scholte et al. (2016)
	Tailor outreach for collaboration to a broad swath of the public, including relevant private stakeholders to prevent or alleviate conflicts	Koutrakis et al. (2011), Kuo et al. (2015)
**Meaningful and active** Meaningful participation gives real voice and decision-making power to the public (van den Hoek et al. 2014), while personal experiences can strongly influence attitudes	Support the establishment of ad hoc local institutions, offices, committees, or citizen-based advisory groups	Myatt et al. (2003a), Davis and Cole (2004), Davenport et al. (2010), Everett and Lamond (2018)
	Explore the use of creative and fit-to-purpose plans for collaboration (e.g. thematic working groups led by informed local stakeholders)	Schmidt et al. (2013), Schernewski et al. (2017)
	Consider interactive, hands-on and experiential participatory activities such as workshops, field trips, or volunteer stewardship programmes	Bihari and Ryan (2012), Schmidt et al. (2013), Chou (2016)
**Educational and capacity-building** Participation and collaboration, such as co-management or stewardship schemes, may require that certain knowledge and skills first be acquired (Barbier 2006; On-prom 2014)	Provide capacity-building when needed in relation to acquisition of co-benefits, for example how to take advantage of nature-based tourism for local businesses	Davenport et al. (2010)
	Consider residents’ personal experiences (e.g. past environmental/risk management)	Bihari and Ryan (2012)
	Involve relevant institutions, fostering bi-directional learning to and from citizens	Santoro et al. (2019)

## Discussion

Our review leads to three broad insights.

1. In line with key NbS literature, we find that NbS involve distinct social interactions across project phases compared to traditional grey infrastructure measures for reducing risk. Moreover, the long-term success of NbS consistently relies on a broader range of public acceptance outcomes.
2. Given their reliance on public acceptance, a number of interconnected factors related to the measure itself, the individual, and the societal context are crucial for the success of NbS. These factors are highly contextual in their strength of influence, but broad in their potential applicability and therefore worthy of systematic consideration.
3. Strategies for providing benefits, increasing public awareness of benefits, communicating effectively, and promoting participation and collaboration are suggested for leveraging the identified factors and increasing public acceptance of NbS.

**Fig. 8:**
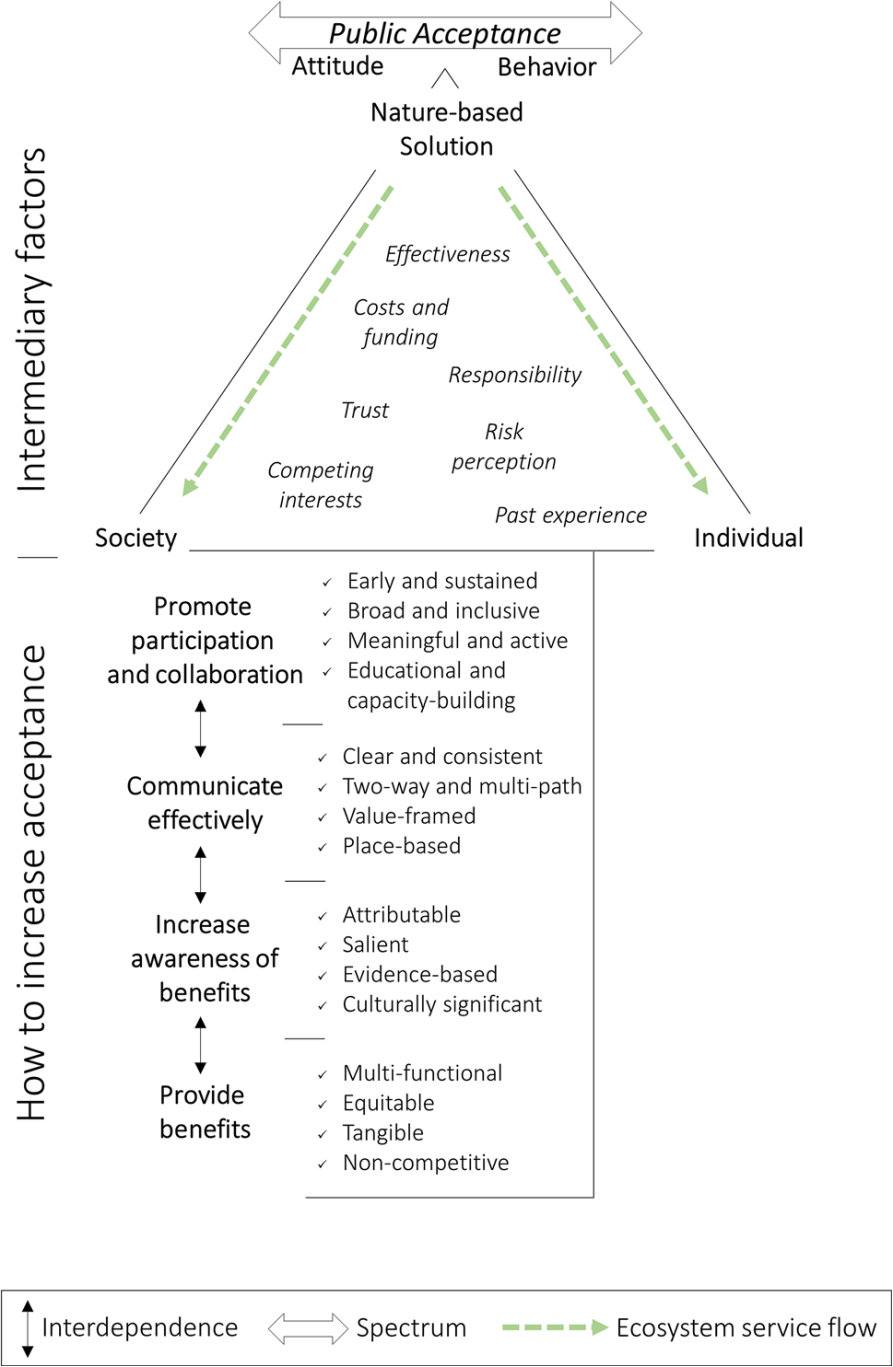
The Public Acceptance of Nature-based Solutions framework (PA-NbS) depicts recommendations and corresponding success criteria. These act on and through contextual factors at the nexus of the individual, the society, and the NbS. Ecosystem services represent the crucial factor of perceived benefits and trade-offs. These flow within this nexus from the NbS and are perceived (or not) by individuals and society. Public acceptance is case-specific, exists on a spectrum, and is manifested by attitudes and behaviours, which also act on each other causally

To provide NbS practitioners and researchers a basis for structured consideration of how to increase public acceptance, we graphically represent the relevant review findings to create the Public Acceptance of Nature-based Solutions framework (PA-NbS) (Fig. 8). The PA-NbS thus provides a starting point for the design and testing of strategies to increase NbS acceptance. When possible, the four interdependent principal recommendations and four corresponding success criteria that form the base of the framework should be met (taken from Table 4). Moving from the bottom to the top of the framework, these recommendations act on and are modulated by influencing factors for public acceptance within the nexus of the individual, the society, and the NbS. The factors provided are illustrative examples (taken from Table 3) positioned within the triangle in accordance to their relevance to the individual, society, and the NbS. The flow of ecosystem services from the NbS to individuals and society represents the most commonly cited underlying factor for public acceptance—perceived benefits. In the framework, if the recommendations are acted on and appropriately adapted to the context found at this nexus, they lead to public acceptance of the NbS.

Acceptance is manifested in positive public attitudes and/or behaviours. Attitude can shape behaviour just as behaviour can shape attitude (Spence and Pidgeon 2009), but the precise definition of public acceptance should be case-specific and ideally co-determined using goals and indicators with the public itself.

We mostly find a high degree of overlap between the recommendations in the PA-NbS that are directly related to the measure (providing benefits and promoting participation and collaboration) and NbS principals (Cohen-Shacham et al. 2016) and the Global Standard for NbS outlined by the IUCN (IUCN 2020). Principle 4, for example, calls for producing “societal benefits in a fair and equitable way in a manner that promotes transparency and broad participation” (Cohen-Shacham et al. 2016, p.6), while Criterion 6 of the Global Standard provides indicators for assessing whether benefits and trade-offs are equitable (IUCN 2020). Stakeholder involvement, recognizing and limiting trade-offs, ensuring public understanding and incorporating public values are all key elements of the documents. However, whether benefits are tangible to the public (or not) is lacking. Similarly, the principles and Global Standard fail to emphasize the importance of not only providing benefits, but also promoting awareness of them. Increasing awareness was highlighted as one of four key overarching recommendation in the reviewed literature. Because NbS rely more heavily on public acceptance for success than grey measures and are often perceived as novel, complex, and value-laden, we recommend that the criteria regarding increasing awareness of benefits be addressed in the core principles.

The importance of aesthetics of NbS has been demonstrated in other contexts and should be a point of emphasis in designs and planning, as well as communicating co-benefits and trust (Frantzeskaki 2019). Our findings also corroborate those of a recent review on NbS perceptions by Han and Kuhlicke (2019). In particular, they also find a focus in the literature on co-benefits, risk reduction efficacy, participation, environmental attitudes, and uncertainty surrounding NbS for forming perceptions. Likewise, they discuss the seemingly negative association between threat-appraisal and trust in NbS. This lends credence to the importance of risk (in)tolerance as well as perceived effectiveness in relation to acceptance as presented in our RP-MAM model (see the ‘Risk Perception’ section). The RP-MAM model should be considered a first step towards understanding the interconnections among the key factors of risk perception, risk (in)tolerance, perceived effectiveness, and perceived co-benefits in relation to NbS acceptance. The model is currently being tested with data from NbS sites in the OPERANDUM project.^2^

Societal acceptance and sustained success of NbS is not limited to the perception of citizens living in and around NbS, but also determined by a host of legal, governmental, economic and technical factors (Nesshöver et al. 2017; Wamsler et al. 2019). Some of the factors identified in our review exist within such spheres and could be difficult to act on. For example, costs and funding and effectiveness of the measure may be constrained by non-negotiable requirements. However, even practical constraints and quantifiable characteristics are perceived differently and can influence attitudinal and behavioural public acceptance.

Although participation is generally desirable, it may be inappropriate in contexts where decisions have necessarily already been made, past failures have occurred, insufficient resources are available (e.g. for capacity-building), or there is no civic culture. Here, effective communication and consultation may form the basis of more appropriate goals (Reed et al. 2018). Additionally, public engagement can be risky and not always beneficial, depending particularly on the history, flexibility, and capacities of the institutions involved (Wamsler et al. 2019). This underscores the idea that there is effective and ineffective public engagement, and ineffective engagement may lead to worse outcomes than no engagement at all.

Positive attitudes often do not lead to positive behaviours and behavioural motivators may differ greatly (Wachinger et al. 2013). However, to disaggregate the factors on this basis would require a larger dataset and more experimental evidence in the literature. The factors we identify are relevant for promoting positive attitudes and behaviours, since the success of NbS projects often relies on both and they are interconnected. Indeed, increasing awareness of benefits and fostering engagement are key considerations for behaviour change, which may or may not be mediated by effects on attitude change (Spence and Pidgeon 2009). For broad practicality, the factors and recommendations are therefore useful as a starting point for research to determine their relevance, strength and specific contextual characteristics.

Such studies should follow the principle of segmentation, recognizing that the public is not a homogeneous entity. Contradictory public values should be identified since their interplay is key for acceptance (Reed et al. 2018). For example, Scholte et al. (2016) found that biodiversity was a more important factor for farmers than other residents. Several studies also highlight that aesthetics, although important, is subjective among members of the same public (Myatt et al. 2003b; Evans et al. 2017). Using social norms can be a powerful motivator but relevant norms must already exist and this strategy has been shown to backfire depending on in- and out-group dynamics (Bicchieri and Dimant 2019). Likewise, the use of economic incentives may be very effective for some, but have negative externalities such as competing with altruistic or moral motivation (Beery 2018) or raising expectations too high for others (Biswas et al. 2009).

## Conclusions

Using nature to address societal challenges like risk from natural hazards is often highly effective and can deliver a wide range of co-benefits. However, the approach is still perceived as novel compared to traditional grey measures, common for practitioners and the public to rely on in contexts of risk. In many cases, public acceptance of NbS for risk reduction will have to be earned. Along with effectiveness and co-benefits, we identified a number of factors that can influence public perceptions and be leveraged by practitioners and researchers to encourage greater acceptance. Generic education campaigns are a popular recommendation for increasing awareness of benefits. However, it has become increasingly obvious that the presentation of scientific evidence alone can have a very weak influence on public attitudes and behaviour. Further research into alternative approaches to leveraging these factors for acceptance is needed. Moreover, efforts towards establishing principles and standards for NbS should be accompanied by more research into interactions among individuals, societies and NbS for risk reduction. Public perceptions determine acceptance, which is crucial for the success and continued uptake of NbS.

## Supplementary Information

Below is the link to the electronic supplementary material.

Supplementary material 1 (PDF 488 kb)

## Footnotes

1 Other studies, however, indicate low acceptance of depolderization in the U.K. (e.g. Esteves and Thomas 2014).

2 www.operandum-project.eu.

## References

[CR1] Abbas A, Amjath-Babu T, Kächele H, Müller K (2016). Participatory adaptation to climate extremes: An assessment of households’ willingness to contribute labor for flood risk mitigation in Pakistan. Journal of Water and Climate Change.

[CR2] Badola R, Hussain SA (2005). Valuing ecosystem functions: An empirical study on the storm protection function of Bhitarkanika mangrove ecosystem, India. Environmental Conservation.

[CR3] Badola R, Barthwal S, Hussain SA (2011). Attitudes of local communities towards conservation of mangrove forests: A case study from the east coast of India. Estuarine, Coastal and Shelf Science.

[CR4] Barbier EB (2006). Natural barriers to natural disasters: Replanting mangroves after the tsunami. Frontiers in Ecology and the Environment.

[CR5] Beery, T. 2018. Engaging the Private Homeowner: Linking Climate Change and Green Stormwater Infrastructure. *Sustainability* 10. Multidisciplinary Digital Publishing Institute: 4791.

[CR6] Bicchieri, C., and E. Dimant. 2019. Nudging with care: The risks and benefits of social information. *Public Choice*.

[CR7] Bihari M, Ryan R (2012). Influence of social capital on community preparedness for wildfires. Landscape and Urban Planning.

[CR8] Biswas SR, Mallik AU, Choudhury JK, Nishat A (2009). A unified framework for the restoration of Southeast Asian mangroves—bridging ecology, society and economics. Wetlands Ecology and Management.

[CR9] Brandolini SMD, Disegna M (2015). ICZM and WTP of stakeholders for beach conservation: Policymaking suggestions from an Italian case study. Tourism Economics.

[CR10] Brink E, Wamsler C (2019). Citizen engagement in climate adaptation surveyed: The role of values, worldviews, gender and place. Journal of cleaner production.

[CR11] Brundtland, G.H., M. Khalid, S. Agnelli, S. Al-Athel, and B. Chidzero. 1987. Our common future. *New York*.

[CR12] Bubeck P, Botzen W, Suu L, Aerts J (2012). Do flood risk perceptions provide useful insights for flood risk management? Findings from central Vietnam. Journal of Flood Risk Management.

[CR13] Buchecker, M., S. Menzel, and R. Home. 2013. How much does participatory flood management contribute to stakeholders’ social capacity building? Empirical findings based on a triangulation of three evaluation approaches. *Natural Hazards and Earth System Sciences* 13. Copernicus GmbH: 1427–1444.

[CR14] Buchecker M, Ogasa DM, Maidl E (2015). How well do the wider public accept integrated flood risk management? An empirical study in two Swiss Alpine valleys. Environmental Science & Policy.

[CR15] Buijs AE (2009). Public support for river restoration. A mixed-method study into local residents’ support for and framing of river management and ecological restoration in the Dutch floodplains. Journal of Environmental Management.

[CR16] Calvello M, Papa MN, Pratschke J, Crescenzo MN (2016). Landslide risk perception: a case study in Southern Italy. Landslides.

[CR17] Chen C, Wang Y, Jia J (2018). Public perceptions of ecosystem services and preferences for design scenarios of the flooded bank along the Three Gorges Reservoir: Implications for sustainable management of novel ecosystems. Urban Forestry & Urban Greening.

[CR18] Chou R-J (2016). Achieving successful river restoration in dense urban areas: Lessons from Taiwan. Sustainability.

[CR19] Chowdhury MR (2002). The Impact of Greater Dhaka Flood Protection Project’ (GDFPP) on local living environment-the attitude of the floodplain residents. Natural Hazards.

[CR20] Cohen-Shacham E, Walters G, Janzen C, Maginnis S (2016). Nature-based solutions to address global societal challenges.

[CR21] Davenport MA, Bridges CA, Mangun JC, Carver AD, Williard KW, Jones EO (2010). Building local community commitment to wetlands restoration: A case study of the Cache River wetlands in southern Illinois, USA. Environmental Management.

[CR22] Davis G, Cole K (2004). Community involvement in coast protection at Lyme Regis, UK. Proceedings of the Institution of Civil Engineers-Municipal Engineer.

[CR23] Ding L, Ren X, Gu R, Che Y (2019). Implementation of the “sponge city” development plan in China: An evaluation of public willingness to pay for the life-cycle maintenance of its facilities. Cities.

[CR24] Drake B, Smart JC, Termansen M, Hubacek K (2013). Public preferences for production of local and global ecosystem services. Regional Environmental Change.

[CR25] Esteves LS, Thomas K (2014). Managed realignment in practice in the UK: Results from two independent surveys. Journal of Coastal Research.

[CR26] Commission European (2000). Directive 2000/60/EC of the European Parliament and of the Council of 23 October 2000 establishing a framework for community action in the field of water policy. Official Journal of the European Communities L.

[CR27] Evans AJ, Garrod B, Firth LB, Hawkins SJ, Morris-Webb ES, Goudge H, Moore PJ (2017). Stakeholder priorities for multi-functional coastal defence developments and steps to effective implementation. Marine Policy.

[CR28] Everett G, Lamond JE (2018). Considering the value of community engagement for (co-) producing blue-green infrastructure. WIT Transactions on the Built Environment.

[CR29] Everett G, Lamond J, Morzillo AT, Matsler AM, Chan FKS (2018). Delivering Green Streets: an exploration of changing perceptions and behaviours over time around bioswales in Portland, Oregon. Journal of Flood Risk Management.

[CR30] Faivre N, Fritz M, Freitas T, de Boissezon B, Vandewoestijne S (2017). Nature-based solutions in the EU: Innovating with nature to address social, economic and environmental challenges. Environmental Research.

[CR31] Fordham M, Tunstall S, Penning-Rowsell E (1991). Choice and preference in the Thames floodplain: The beginnings of a participatory approach?. Landscape and Urban Planning.

[CR32] Frantzeskaki N (2019). Seven lessons for planning nature-based solutions in cities. Environmental Science & Policy.

[CR33] Fuchs S, Karagiorgos K, Kitikidou K, Maris F, Paparrizos S, Thaler T (2017). Flood risk perception and adaptation capacity: A contribution to the socio-hydrology debate. Hydrology and Earth System Sciences.

[CR34] Geaves LH, Penning-Rowsell EC (2015). Flood risk management as a public or a private good, and the implications for stakeholder engagement. Environmental Science & Policy.

[CR35] Ghanbarpour MR, Saravi MM, Salimi S (2014). Floodplain inundation analysis combined with contingent valuation: Implications for sustainable flood risk management. Water Resources Management.

[CR36] Godschalk D, Brody S, Burby R (2003). Public participation in natural hazard mitigation policy formation: Challenges for comprehensive planning. Journal of Environmental Planning and Management.

[CR37] Goeldner-Gianella L, Bertrand F, Oiry A, Grancher D (2015). Depolderisation policy against coastal flooding and social acceptability on the French Atlantic coast: The case of the Arcachon Bay. Ocean & Coastal Management.

[CR38] De Groot M (2012). Exploring the relationship between public environmental ethics and river flood policies in western Europe. Journal of Environmental Management.

[CR39] De Groot, M., and W.T. de Groot. 2009. “Room for river” measures and public visions in the Netherlands: A survey on river perceptions among riverside residents. *Water Resources Research* 45.

[CR40] Haddaway N, Macura B, Whaley P, Pullin A (2017). ROSES for systematic review protocols. Version1.0.

[CR41] Han S, Kuhlicke C (2019). Reducing hydro-meteorological risk by nature-based solutions: What do we know about people’s perceptions?. Water.

[CR42] Herringshaw CJ, Thompson JR, Stewart TW (2010). Learning about restoration of urban ecosystems: A case study integrating public participation, stormwater management, and ecological research. Urban Ecosystems.

[CR43] Van den Hoek, R., M. Brugnach, J. Mulder, and A. Hoekstra. 2014. Uncovering the origin of ambiguity in nature-inclusive flood infrastructure projects. *Ecology and Society* 19.

[CR44] Holcombe EA, Berg E, Smith S, Anderson MG, Holm-Nielsen N (2018). Does participation lead to ongoing infrastructure maintenance? Evidence from Caribbean landslide mitigation projects. The Journal of Development Studies.

[CR45] Holstead K, Kenyon W, Rouillard J, Hopkins J, Galán-Diaz C (2017). Natural flood management from the farmer’s perspective: Criteria that affect uptake. Journal of Flood Risk Management.

[CR46] Hoque MM, Siddique MA (1995). Flood control projects in Bangladesh: Reasons for failure and recommendations for improvement. Disasters.

[CR47] Howgate OR, Kenyon W (2009). Community cooperation with natural flood management: A case study in the Scottish Borders. Area.

[CR48] [IUCN] International Union for Conservation of Nature and Natural Resources. 2020. Global Standard for Nature-based Solutions. A user-friendly framework for the verification, design and scaling up of NbS. First edition. Gland, Switzerland: IUCN. 10.2305/IUCN.CH.2020.08.en.

[CR49] Kabisch, N., N. Frantzeskaki, S. Pauleit, S. Naumann, M. Davis, M. Artmann, D. Haase, S. Knapp, et al. 2016. Nature-based solutions to climate change mitigation and adaptation in urban areas: perspectives on indicators, knowledge gaps, barriers, and opportunities for action. *Ecology and Society* 21.

[CR50] Kienker S, Coleman R, Morris R, Steinberg P, Bollard B, Jarvis R, Alexander K, Strain E (2018). Bringing harbours alive: assessing the importance of eco-engineered coastal infrastructure for different stakeholders and cities. Marine Policy.

[CR51] Kim T-G, Petrolia DR (2013). Public perceptions of wetland restoration benefits in Louisiana. ICES Journal of Marine Science.

[CR52] Koutrakis E, Sapounidis A, Marzetti S, Marin V, Roussel S, Martino S, Fabiano M, Paoli C (2011). ICZM and coastal defence perception by beach users: Lessons from the Mediterranean coastal area. Ocean & Coastal Management.

[CR53] Kuo Y-L, Chang C-C, Li H-C (2015). Lulling effect of public flood protection: Case of Benhe community in Kaohsiung during Typhoon Fanapi. Natural Hazards Review.

[CR54] Lara A, Sauri D, Ribas A, Pavón D (2010). Social perceptions of floods and flood management in a Mediterranean area (Costa Brava, Spain). Natural Hazards and Earth System Sciences.

[CR55] [MEA] Millenium Ecosystem Assessment (2005). Ecosystems and human well-being: Health synthesis.

[CR56] Mees, H., A. Crabbé, M. Alexander, M. Kaufmann, S. Bruzzone, L. Lévy, and J. Lewandowski. 2016. Coproducing flood risk management through citizen involvement: Insights from cross-country comparison in Europe. *Ecology and Society* 21

[CR57] Miller SM, Montalto FA (2019). Stakeholder perceptions of the ecosystem services provided by Green Infrastructure in New York City. Ecosystem Services.

[CR58] Myatt LB, Scrimshaw MD, Lester JN (2003). Public perceptions and attitudes towards a forthcoming managed realignment scheme: Freiston Shore, Lincolnshire, UK. Ocean and Coastal Management.

[CR59] Myatt LB, Scrimshaw MD, Lester JN (2003). Public perceptions and attitudes towards an established managed realignment scheme: Orplands, Essex, UK. Journal of Environmental Management.

[CR60] Myatt-Bell LB, Scrimshaw MD, Lester JN, Potts JS (2002). Public perception of managed realignment: Brancaster West Marsh, North Norfolk, UK. Marine Policy.

[CR61] Neef, A., P. Elstner, and I. Schad. 2014. The interplay between collective action, individual strategies and state intervention in mitigating flood disasters in the uplands of North Thailand and Northwest Vietnam. In *Risks and Conflicts: Local Responses to Natural Disasters*, 109–130. Emerald Group Publishing Limited.

[CR62] Nesshöver C, Assmuth T, Irvine KN, Rusch GM, Waylen KA, Delbaere B, Haase D, Jones-Walters L (2017). The science, policy and practice of nature-based solutions: An interdisciplinary perspective. Science of the Total Environment.

[CR63] Nguyen T, Van Tam N, Parnell KE (2015). Community perspectives on an internationally funded mangrove restoration project: Kien Giang province, Vietnam. Ocean & Coastal Management.

[CR64] On-prom, S. 2014. Community-based mangrove forest management in Thailand: key lesson learned for environmental risk management. In *Sustainable Living with Environmental Risks*, 87–96.

[CR65] Otto A, Hornberg A, Thieken A (2018). Local controversies of flood risk reduction measures in Germany. An explorative overview and recent insights. Journal of Flood Risk Management.

[CR66] Pueyo-Ros J, Ribas A, Fraguell RM (2018). A cultural approach to wetlands restoration to assess its public acceptance. Restoration Ecology.

[CR67] Rambonilaza T, Joalland O, Brahic E (2016). Landowner’s perception of flood risk and preventive actions in estuarine environment: An empirical investigation. Journal of Environmental Management.

[CR68] Reed MS (2008). Stakeholder participation for environmental management: A literature review. Biological Conservation.

[CR69] Reed MS, Vella S, Challies E, de Vente J, Frewer L, Hohenwallner-Ries D, Huber T, Neumann RK (2018). A theory of participation: What makes stakeholder and public engagement in environmental management work?. Restoration Ecology.

[CR70] Reilly K, Adamowski J, John K (2018). Participatory mapping of ecosystem services to understand stakeholders’ perceptions of the future of the Mactaquac Dam, Canada. Ecosystem Services.

[CR71] Renaud FG, Sudmeier-Rieux K, Estrella M, Nehren U (2016). Ecosystem-based disaster risk reduction and adaptation in practice.

[CR72] Roca E, Villares M (2012). Public perceptions of managed realignment strategies: The case study of the Ebro Delta in the Mediterranean basin. Ocean and Coastal Management.

[CR73] Ryan RL, Wamsley MB (2008). Public perceptions of wildfire risk and forest management in the Central Pine Barrens of Long Island (USA). The Australasian Journal of Disaster and Trauma Studies.

[CR74] Saengsupavanich C (2012). Detached breakwaters: Communities’ preferences for sustainable coastal protection. Journal of Environmental Management.

[CR75] Santoro S, Pluchinotta I, Pagano A, Pengal P, Cokan B, Giordano R (2019). Assessing stakeholders’ risk perception to promote Nature Based Solutions as flood protection strategies: The case of the Glin\vs\vcica river (Slovenia). Science of the Total Environment.

[CR76] Sarzynski, A., and P. Cavaliere. 2018. Public participation in planning for community management of natural hazards. In *Oxford Research Encyclopedia of Natural Hazard Science*.

[CR77] Schaich H (2009). Local residents’ perceptions of floodplain restoration measures in Luxembourg’s Syr Valley. Landscape and Urban Planning.

[CR78] Schernewski G, Schumacher J, Weisner E, Donges L (2017). A combined coastal protection, realignment and wetland restoration scheme in the southern Baltic: Planning process, public information and participation. Journal of Coastal Conservation.

[CR79] Schmidt L, Gomes C, Guerreiro S, O’Riordan T (2013). Are we all on the same boat? The challenge of adaptation facing Portuguese coastal communities: Risk perception, trust-building and genuine participation. Land Use Policy.

[CR80] Scholte SS, Todorova M, van Teeffelen AJ, Verburg PH (2016). Public support for wetland restoration: What is the link with ecosystem service values?. Wetlands.

[CR81] [CBD] Secretariat of the Convention on Biological Diversity. 2019. *Voluntary Guidelines for the Design and Effective Implementation of Ecosystem*-*based Approaches to Climate Change Adaptation and Disaster risk Reduction and Supplementary Information*. *CBD Technical Series No. 93*.

[CR83] Spence A, Pidgeon N (2009). Psychology, climate change & sustainable bahaviour. Environment Science and Policy for Sustainable Development.

[CR84] Tanaka N, Jinadasa K, Mowjood M, Fasly M (2011). Coastal vegetation planting projects for tsunami disaster mitigation: effectiveness evaluation of new establishments. Landscape and Ecological Engineering.

[CR85] Terpstra T, Gutteling JM, Geldof GD, Kappe LJ (2006). The perception of flood risk and water nuisance. Water Science and Technology.

[CR86] Touili N, Baztan J, Vanderlinden J-P, Kane IO, Diaz-Simal P, Pietrantoni L (2014). Public perception of engineering-based coastal flooding and erosion risk mitigation options: Lessons from three European coastal settings. Coastal Engineering.

[CR87] Trialfhianty TI, Suadi (2017). The role of the community in supporting coral reef restoration in Pemuteran, Bali, Indonesia. Journal of coastal conservation.

[CR88] Triyanti A, Chu E (2018). A survey of governance approaches to ecosystem-based disaster risk reduction: Current gaps and future directions. International Journal of Disaster Risk Reduction.

[CR89] Triyanti A, Bavinck M, Gupta J, Marfai MA (2017). Social capital, interactive governance and coastal protection: The effectiveness of mangrove ecosystem-based strategies in promoting inclusive development in Demak, Indonesia. Ocean and Coastal Management.

[CR90] [UNISDR] United Nations International Strategy for Disaster Reduction. 2015. Sendai Framework for Disaster Risk Reduction 2015-2030. http://www.unisdr.org/files/43291_sendaiframeworkfordrren.pdf.

[CR91] Van der Vegt R (2018). A literature review on the relationship between risk governance and public engagement in relation to complex environmental issues. Journal of Risk Research.

[CR92] Verbrugge LN, Ganzevoort W, Fliervoet JM, Panten K, van den Born RJ (2017). Implementing participatory monitoring in river management: The role of stakeholders’ perspectives and incentives. Journal of Environmental Management.

[CR93] Wachinger G, Renn O, Begg C, Kuhlicke C (2013). The risk perception paradox-implications for governance and communication of natural hazards. Risk analysis : an official publication of the Society for Risk Analysis.

[CR94] Wamsler, C., J. Alkan-Olsson, H. Björn, H. Falck, H. Hanson, T. Oskarsson, E. Simonsson, and F. Zelmerlow. 2019. Beyond participation: when citizen engagement leads to undesirable outcomes for nature-based solutions and climate change adaptation. *Climatic Change*: 1–20.

[CR95] Wüstenhagen R, Wolsink M, Bürer MJ (2007). Social acceptance of renewable energy innovation: An introduction to the concept. Energy policy.

