# Supplementary material for: A review of public acceptance of nature-based solutions: The ‘why’, ‘when’, and ‘how’ of success for disaster risk reduction measures
Source: Ambio. 2021 Feb 19;50(8):1552–73. doi: 10.1007/s13280-021-01502-4 (PMC8249538; doi:10.1007/s13280-021-01502-4)
Supplement: Supplementary file 1 — Supplementary material 1 (PDF 488 kb) [file 13280_2021_1502_MOESM1_ESM.pdf]

Ambio Electronic Supplementary Material
This supplementary material has not been peer reviewed.

Title: **A review of public acceptance of nature-based solutions: the 'why', 'when', and 'how' of success for disaster risk reduction measures**

Supplementary Tables

Table S1. Terminology used to describe nature-based solutions (NbS) in the 65 reviewed NbS articles. Terms are grouped into classes based primarily on the authors' own description of the NbS and are listed in order from highest frequency to lowest frequency.

Term used	Reference
**Ecological restoration (n=17)**	
Mangrove restoration	(Badola and Hussain 2005)
Mangrove restoration	(Biswas et al. 2008)
Mangrove restoration	(Gilman and Ellison 2007)
Mangrove restoration	(Iftekhar and Takama 2008)
Mangrove replanting/restoration	(Barbier 2006)
Mangrove conservation	(Badola et al. 2012)
Wetland restoration	(Kim and Petrolia 2013)
Wetland restoration	(Scholte et al. 2016)
Wetland restoration	(Davenport et al. 2010)
Wetland restoration	(Pueyo-Ros et al. 2019)
Wetland restoration and conservation	(Wang et al. 2018)
Floodplain restoration	(Barthélémy and Armani 2015)
Floodplain restoration	(Schaich 2009)
River restoration	(Buijs 2009)
Coral reef restoration	(Trialfhianty and others 2017)
Ecological restoration	(Herringshaw et al. 2010)
Coastal vegetation planting	(Tanaka et al. 2011)
**Risk and ecosystem management (n=15)**	
Flood risk management	(Buchecker et al. 2013)
Flood risk management	(Geaves and Penning-Rowsell 2015)
Natural flood management (NFM)	(Holstead et al. 2017)
Natural flood management (NFM)	(Howgate and Kenyon 2009)
Flood management strategies	(Vávra et al. 2017)
Integrated coastal zone management (ICZM)	(Koutrakis et al. 2011)
Integrated coastal zone management (ICZM)	(Brandolini and Disegna 2015)
Integrated flood risk management	(Buchecker et al. 2016)
Coastal zone management	(Jones et al. 2013)
Urban storm-water management	(Kuo et al. 2015)
'Blue-Green' approaches to Flood Risk Management (BG-FRM)	(Everett and Lamond 2014)
Wetland management	(Rambonilaza et al. 2016)
Risk management initiatives - coastal resilience planning	(Bostick et al. 2017)
Community-based mangrove management (CBMM)	(Damastuti and de Groot 2017)
Community-based natural resource management (CBNRM)	(On-prom 2014)
**Green and blue-green infrastructure (n=13)**	

Green infrastructure	(Beery 2018)
Green infrastructure	(Chou 2016)
Green infrastructure	(Dhakal and Chevalier 2017)
Green infrastructure	(Duan et al. 2018)
Green infrastructure	(Miller and Montalto 2019)
Conventional or green infrastructure	(Reynaud et al. 2017)
Blue-Green infrastructure (BGI)	(Everett and Lamond 2018)
Blue-Green infrastructure (BGI)	(Everett et al. 2018)
Engineered and natural infrastructure	(Gray et al. 2017)
Sponge city	(Ding et al. 2019)
Sponge city	(Wang et al. 2017)
Multi-functional coastal defence structures	(Evans et al. 2017)
Engineering solutions	(Saengsupavanich 2013)
Managed realignment (n=6)	
Managed realignment	(Esteves and Thomas 2014)
Managed realignment	(L. Myatt et al. 2003)
Managed realignment	(L. B. Myatt et al. 2003)
Managed realignment	(Myatt-Bell et al. 2002)
Managed realignment	(Roca and Villares 2012)
Depolderization	(Goeldner-Gianella et al. 2015)
Risk reduction and mitigation measures (n=6)	
Flood risk reduction measures	(Otto et al. 2018)
Hazard reduction strategies	(Ryan and Wamsley 2008)
Mitigation measures	(McGee 2007)
Wildfire mitigation	(Christianson et al. 2013)
Engineering-based coastal flooding and erosion risk mitigation options	(Touili et al. 2014)
Risk reduction and adaption actions	(Brink and Wamsler 2019)
Ecosystem-based approaches (n=5)	
Eco-engineering	(Kienker et al. 2018)
Eco-engineering	(Nguyen et al. 2015)
Ecosystem-based adaptation	(Carro et al. 2018)
Ecosystem-based approaches	(Triyanti et al. 2017)
Building with Nature (BwN)	(van den Hoek et al. 2014)
Other (n=3)	
Defensible space actions	(Bihari and Ryan 2012)
Tree planting and filling in drainage ditches	(Drake et al. 2013)
Landscape engineering	(Chen et al. 2018)

Table S2. Manifestations and indicators of acceptance identified in the reviewed literature, grouped into generally positive, neutral, or negative associations with acceptance.

	Acceptance manifestations and indicators	Example references
Positive	*Acceptance*	(Buchecker et al. 2015; Dhakal and Chevalier 2017; Everett et al. 2018)
	Buy-in	(Esteves and Thomas 2014)
	Commitment	(Davenport et al. 2010)
	Cooperation	(Howgate and Kenyon 2009)
	Engagement	(Beery 2018; Everett and Lamond 2018)
	Intention	(Bubeck et al. 2012)
	Interest	(Biswas et al. 2009; Herringshaw et al. 2010)
	Involvement	(Buchecker et al. 2013; Nguyen et al. 2015)
	Participation	(Godschalk et al. 2003; Fuchs et al. 2017)
	Satisfaction	(Jones et al. 2014)
	Support	(Geaves and Penning-Rowsell 2015; Chou 2016; Kienker et al. 2018)
	Uptake	(Holcombe and Anderson 2010)
	Willing to collaborate	(Bihari and Ryan 2012)
	Willingness to pay	(Ghanbarpour et al. 2014; Goeldner-Gianella et al. 2015)
Neutral	*Attitude*	(Holstead et al. 2017; Chen et al. 2018; Duan et al. 2018)
	Behaviour	(Everett and Lamond 2014)
	Perception	(Gray et al. 2017; Duan et al. 2018)
	Preferences	(Fordham et al. 1991; Lara et al. 2010; Boyer-Villemaire et al. 2014)
	Valuation	(Rasid et al. 1996)
Negative	*Aversion*	(Gray et al. 2017)
	Conflict	(Myatt et al. 2003a; Roca and Villares 2012; Geaves and Penning-Rowsell 2015)
	Lack of participation	(Biswas et al. 2009)
	Opposition	(de Groot and de Groot 2009)
	Protest	(Buijs 2009; Schernewski et al. 2017)
	Rejection	(Saengsupavanich 2013; Goeldner-Gianella et al. 2015)
	Resistance	(Davis and Cole 2004)
	Tension	(Otto et al. 2018)

Table S3. Influencing factors for public acceptance grouped by relation to the measure, the individual, or the society. Within these groupings, the factors are listed from highest frequency to lowest frequency considering all the articles (n=99; including articles describing NbS [n=65], grey measures [n=28], and two or more measures [n=6]). The second column (green) shows the number and percentage of NbS articles (out of the 65 total) that reference each factor in relation to public acceptance. The third column (grey) replicates this for articles describing grey measures. A factor's row is highlighted in green if the factor a) occurs in n>=10 total articles and b) the percentage of NbS articles that reference it is at least double the percentage of grey articles that reference it. An example is provided in the footnote of the table.

Influencing factors for public acceptance	Frequency of articles describing NbS or grey measures that reference each factor		Example references	
NbS/Grey/Two or more (n=99)	NbS (n=65)	Grey (n=28)	NbS	Grey
Factors related to the measure (n=13)				
Benefits and trade-offs of measure[1] 62 (63%)	48 (74%)	10 (36%)	(Buijs 2009; Barthélémy and Armani 2015; Evans et al. 2017)	(McCarthy and Penning-Rowsell 2008; Holcombe et al. 2018; Reilly et al. 2018)
Effectiveness of measure for risk reduction 37 (37%)	31 (48%)	4 (14%)	(Badola and Hussain 2005; Howgate and Kenyon 2009; Carro et al. 2018)	(Abbas et al. 2016; Wedawatta et al. 2016; Verbrugge et al. 2017)
Costs and funding 13 (13%)	12 (18%)	1 (4%)	(Myatt et al. 2003a; Beery 2018; Brink and Wamsler 2019)	(Ghanbarpour et al. 2014)
Financial compensation or incentives 6 (6%)	5 (8%)	1 (4%)	(Buchecker et al. 2013; Damastuti and de Groot 2017; Otto et al. 2018)	(Abbas et al. 2016)
Effectiveness of communication and collaboration 6 (6%)	4 (6%)	1 (4%)	(Howgate and Kenyon 2009; Otto et al. 2018; Ding et al. 2019)	(Calvello et al. 2016)
Uncertainty and complexity of measure 6 (6%)	3 (5%)	2 (7%)	(Schernewski et al. 2017; Brink and Wamsler 2019)	(Godschalk et al. 2003; Reilly et al. 2018)
Equity of costs and benefits 5 (5%)	5 (8%)	0	(Drake et al. 2013; Geaves and Penning-Rowsell 2015; Otto et al. 2018)	N/A

Health and safety concerns 3 (3%)	3 (5%)	0	(Ryan and Wamsley 2008; van den Hoek et al. 2014; Everett et al. 2018)	N/A
Implementation / construction externalities 3 (3%)	2 (3%)	1 (4%)	(Myatt et al. 2003a; Saengsupavanich 2013)	(Myatt et al. 2003a; Saengsupavanich 2012; Saengsupavanich 2013)
Past institutional outreach 3 (3%)	2 (3%)	1 (4%)	(On-prom 2014; Buchecker et al. 2015)	(Holcombe and Anderson 2010)
Past effectiveness of DRR measures 3 (3%)	2 (3%)	1 (4%)	(Badola et al. 2011; Buchecker et al. 2015)	(Verbrugge et al. 2017)
Media coverage 3 (3%)	3 (5%)	0	(Schernewski et al. 2017; Miller and Montalto 2019)	N/A
Duration of implementation 1 (1%)	1 (2%)	0	(Schernewski et al. 2017)	N/A
Factors related to the individual (n=15)				
Risk perception of natural hazards 33 (33%)	18 (28%)	12 (43%)	(McGee 2007; Kim and Petrolia 2013; Holstead et al. 2017)	(Fordham et al. 1991; Holcombe et al. 2018; Houston et al. 2019)
Awareness and understanding of measure 20 (20%)	15 (23%)	3 (11%)	(Ryan and Wamsley 2008; Schernewski et al. 2017; Kienker et al. 2018)	(Hoque and Siddique 1995; Figueiredo et al. 2009; Neef et al. 2014)
Awareness of benefits 17 (17%)	13 (20%)	4 (14%)	(Nguyen et al. 2015; Scholte et al. 2016; Everett and Lamond 2018)	(Saengsupavanich 2012; Abbas et al. 2016; Holcombe et al. 2018)
Responsibility for measure 17 (17%)	14 (22%)	3 (11%)	(Touili et al. 2014; Rambonilaza et al. 2016; Everett et al. 2018)	(Scally and Wescott 2011; Neef et al. 2014; Fuchs et al. 2017)
Participation 11 (11%)	9 (14%)	2 (7%)	(Howgate and Kenyon 2009; Herringshaw et al. 2010; On-prom 2014)	(Fordham et al. 1991; Davis and Cole 2004)
Fatalist or agentic perspective 8 (8%)	5 (8%)	3 (11%)	(Bihari and Ryan 2012; Everett et al. 2018; Brink and Wamsler 2019)	(Schmidt et al. 2013; Abbas et al. 2016; Fuchs et al. 2017)
Past experience with hazard 8 (8%)	5 (8%)	3 (11%)	(Badola et al. 2011; Bihari and Ryan 2012; Brink and Wamsler 2019)	(Godschalk et al. 2003; Lara et al. 2010; Ghanbarpour et al. 2014)

Age of individual 7 (7%)	5 (8%)	2 (7%)	(Myatt et al. 2003a; Schernewski et al. 2017; Duan et al. 2018)	(Schmidt et al. 2013; Abbas et al. 2016)
Expectations of measure 6 (6%)	5 (8%)	1 (4%)	(Biswas et al. 2009; Chou 2016; Schernewski et al. 2017)	(Verbrugge et al. 2017)
Perceived inclusion or exclusion 5 (5%)	5 (8%)	0	(Triyanti et al. 2017; Everett et al. 2018; Everett et al. 2018; Miller and Montalto 2019)	N/A
Level of education of individual 4 (4%)	4 (6%)	0	(McGee 2007; Badola et al. 2011; Brink and Wamsler 2019)	N/A
Distance from measure 4 (4%)	3 (5%)	1 (4%)	(Myatt et al. 2003b; Schaich 2009; Trialfhianty and Suadi 2017)	(Abbas et al. 2016)
Number of visits to measure 3 (3%)	3 (5%)	0	(Schaich 2009; Reynaud et al. 2017; Duan et al. 2018)	N/A
Income level of individual 2 (2%)	1 (2%)	1 (4%)	(Brink and Wamsler 2019)	(Ghanbarpour et al. 2014)
Mental associations with measure 2 (2%)	1 (2%)	1 (4%)	(Scholte et al. 2016)	(McCarthy and Penning-Rowsell 2008)
Factors related to the society (n=8)				
Place attachment 13 (13%)	8 (12%)	2 (7%)	(Buijs 2009; Bihari and Ryan 2012; Brink and Wamsler 2019)	(Chowdhury 2002; Schmidt et al. 2013)
Trust in responsible party 11 (11%)	7 (11%)	3 (11%)	(Myatt et al. 2003a; Buchecker et al. 2015; Ding et al. 2019)	(Schmidt et al. 2013; Verbrugge et al. 2017)
Competing societal interests 11 (11%)	9 (14%)	2 (7%)	(Barbier 2006; Iftekhar and Takama 2008; Everett et al. 2018)	(Abbas et al. 2016; Holcombe et al. 2018)
Resistance to change and new concepts 7 (7%)	5 (8%)	1 (4%)	(Koutrakis et al. 2011; Schernewski et al. 2017)	(Davis and Cole 2004)
Civic culture and tradition 4 (4%)	3 (5%)	1 (4%)	(Barbier 2006; Gilman and Ellison 2007; Schernewski et al. 2017)	(Schmidt et al. 2013)
Human versus nature perspectives	3	0	(Myatt et al. 2003a; Barthélémy and Armani 2015)	N/A

4 (4%)	(5%)			
**Support of community leader(s)** 2 (2%)	2 (3%)	0	(Damastuti and de Groot 2017; Trialfhianty and Suadi 2017)	N/A
**Social norms** 2 (2%)	2 (3%)	0	(Holstead et al. 2017; Brink and Wamsler 2019)	N/A

[1] 'Benefits and trade-offs of measure' is referenced in 63% (n=62) of all 99 articles. It is referenced in 74% (n=48) of the 65 NbS articles and 36% (n=10) of the 28 grey articles. The remaining four articles of the 62 total in which it is referenced describe two or more measures. It is highlighted in green because a) the total mentions is greater than 10 (62 > 10) and b) the percentage of NbS articles is at least double the percentage of grey articles that reference this factor (74 >= 36*2).

Supplementary Figures

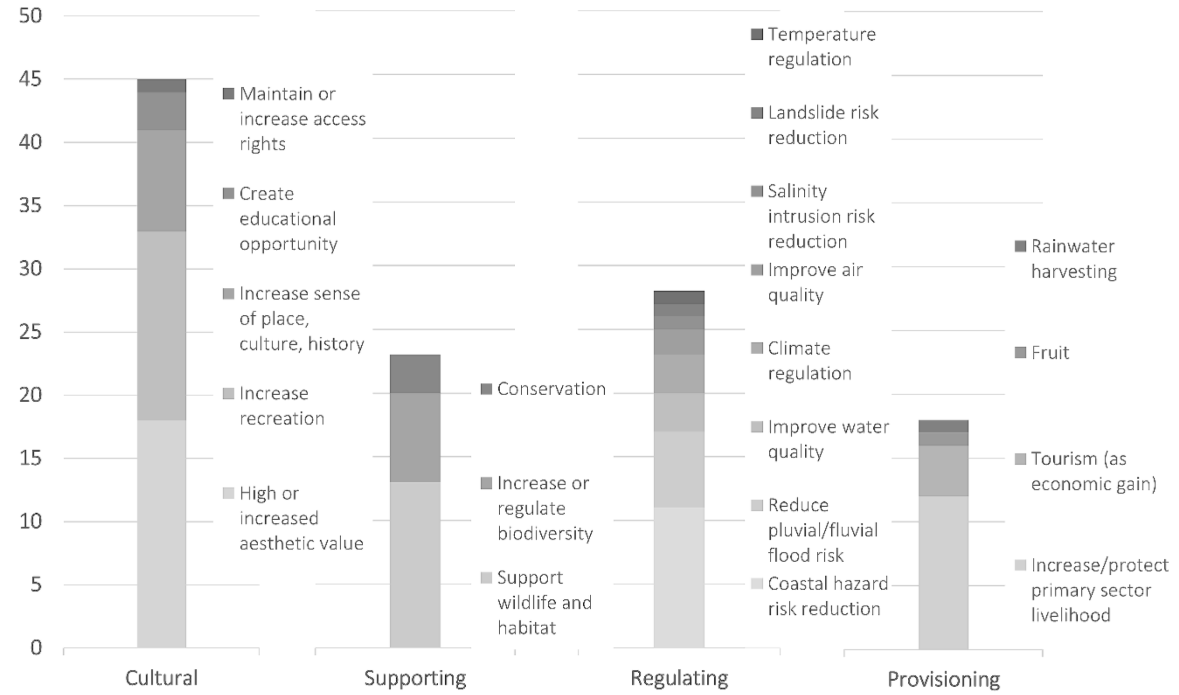

Figure S1. Number of articles in the review that associate public perception of each ecosystem service (cultural, supporting, regulating, and provisioning) with public acceptance of the measures.

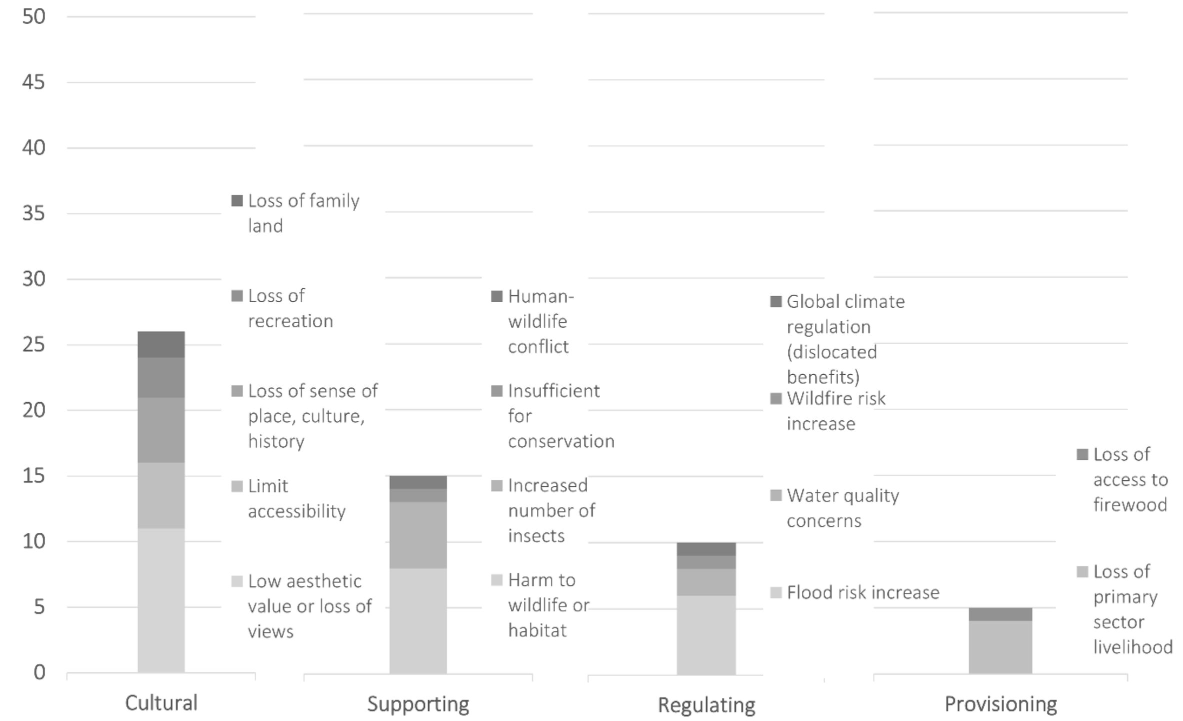

Figure S2. Number of articles in the review that associate public perception of each ecosystem disservice or lack of ecosystem service (cultural, supporting, regulating, and provisioning) with public acceptance of the measures.

Supplementary Text

Text S1. Full search term sequence used in Scopus on May 15, 2019. The search (including automatic screening criteria) yielded 5,900 returns.

TITLE-ABS-KEY ((

(local w/2 accept*) OR (public w/2 accept*) OR (social w/2 accept*) OR (societ* w/2 accept*) OR(stakeholder w/2 accept*) OR (communit* w/2 accept*) OR (individual w/2 accept*) OR(household w/2 accept*) OR(resident w/2 accept*) OR(citizen w/2 accept*) OR (local w/2 reject*) OR (public w/2 reject*) OR (social w/2 reject*) OR(societ* w/2 reject*) OR(stakeholder w/2 reject*) OR(communit* w/2 reject*) OR(individual w/2 reject*) OR(household w/2 reject*) OR(resident w/2 reject*) OR(citizen w/2 reject*) OR (local w/2 apath*) OR (public w/2 apath*) OR(social w/2 apath*) OR(societ* w/2 apath*) OR(stakeholder w/2 apath*) OR(communit* w/2 apath*) OR(individual w/2 apath*) OR(household w/2 apath*) OR (resident w/2 apath*) OR (citizen w/2 apath*) OR (local w/2 fatigue) OR (public w/2 fatigue) OR(social w/2 fatigue*) OR (societ* w/2 fatigue) OR(stakeholder w/2 fatigue) OR(communit* w/2 fatigue) OR(individual w/2 fatigue) OR(household w/2 fatigue) OR(resident w/2 fatigue) OR(citizen w/2 fatigue) OR (local w/2 burnout) OR (public w/2 burnout) OR(social w/2 burnout) OR(societ* w/2 burnout) OR(stakeholder w/2 burnout) OR(communit* w/2 burnout) OR(individual w/2 burnout) OR(household w/2 burnout) OR(resident w/2 burnout) OR(citizen w/2 burnout) OR (local w/2 indifferen*) OR (public w/2 indifferen*) OR (social w/2 indifferen*) OR (societ* w/2 indifferen*) OR(stakeholder w/2 indifferen*) OR (communit* w/2 indifferen*) OR (individual w/2 indifferen*) OR(household w/2 indifferen*) OR (resident w/2 indifferen*) OR (citizen w/2 indifferen*) OR (local w/2 perception) OR (public w/2 perception) OR(social w/2 perception) OR (societ* w/2 perception) OR(stakeholder w/2 perception) OR(communit* w/2 perception) OR(individual w/2 perception) OR(household w/2 perception) OR(resident w/2 perception) OR(citizen w/2 perception) OR (local w/2 participat) OR (public w/2 participat*) OR(social w/2 participat*) OR(societ* w/2 participat*) OR(stakeholder w/2 participat*) OR(communit* w/2 participat*) OR(individual w/2 participat*) OR(household w/2 participat*) OR(resident w/2 participat*) OR(citizen w/2 participat*) OR (local w/2 preference*) OR (public w/2 preference) OR(social w/2 preference) OR(societ* w/2 preference) OR(stakeholder w/2 preference) OR(communit* w/2 preference) OR(individual w/2 preference) OR(household w/2 preference) OR(resident w/2 preference) OR(citizen w/2 preference) OR (local w/2 buy-in) OR (public w/2 buy-in) OR(social w/2 buy-in) OR(societ* w/2 buy-in) OR(stakeholder w/2 buy-in) OR(communit* w/2 buy-in) OR(individual w/2 buy-in) OR(household w/2 buy-in) OR(resident w/2 buy-in) OR(citizen w/2 buy-in) OR (local w/2 involv*) OR (public w/2 involv*) OR(social w/2 involv*) OR(societ* w/2 involv*) OR(stakeholder w/2 involv*) OR(communit* w/2 involv*) OR(individual w/2 involv*) OR(household w/2 involv*) OR(resident w/2 involv*) OR(citizen w/2 involv*) OR (local w/2 engag*) OR (public w/2 engag*) OR(social w/2 engag*) OR(societ* w/2 engag*) OR(stakeholder w/2 engag*) OR(communit* w/2 engag*) OR(individual w/2 engag*) OR(household w/2 engag*) OR(resident w/2 engag*) OR(citizen w/2 engag*) OR (local w/2 "collective action") OR(public w/2 "collective action") OR(social w/2 "collective action") OR(societ* w/2 "collective action") OR(stakeholder w/2 "collective action") OR(communit* w/2 "collective action") OR(individual w/2 "collective action") OR(household w/2 "collective action") OR(resident w/2 "collective action") OR(citizen w/2 "collective action") OR (local w/2 sentiment) OR (public w/2 sentiment) OR(social w/2 sentiment) OR(societ* w/2 sentiment) OR(stakeholder w/2 sentiment) OR(communit* w/2 sentiment) OR(individual w/2 sentiment) OR(household w/2 sentiment) OR(resident w/2 sentiment) OR(citizen w/2 sentiment) OR (local w/2 attitude) OR (public w/2 attitude) OR(social w/2 attitude) OR(societ* w/2 attitude) OR(stakeholder w/2 attitude) OR(communit* w/2 attitude) OR(individual w/2 attitude) OR(household w/2 attitude) OR(resident w/2 attitude) OR(citizen w/2 attitude) OR (local w/2 belief) OR (public w/2 belief) OR(social w/2 belief) OR(societ* w/2 belief) OR(stakeholder w/2 belief) OR(communit* w/2 belief) OR(individual w/2 belief) OR(household w/2 belief) OR(resident w/2 belief) OR(citizen w/2 belief) OR (local w/2 behavio) OR (public w/2 behavio*) OR(social w/2 behavio*) OR(societ* w/2 behavio*) OR(stakeholder w/2 behavio*) OR(communit* w/2 behavio*) OR(individual w/2 behavio*) OR(household w/2 behavio*) OR(resident w/2 behavio*) OR(citizen w/2 behavio*)) **AND**

(resilien* OR drr OR nbs OR "hazard mitigation" OR "hazard adjustment" OR disaster OR "risk mitigation" OR "risk reduction" OR "risk management" OR "risk communication" OR "nature-based solution" OR "eco-engineering" OR "ecological restoration" OR "ecological engineering" OR "forest landscape restoration" OR "ecosystem-based

adaptation" OR "ecosystem-based mitigation" OR "climate adaptation services" OR "ecosystem-based disaster risk reduction" OR "natural infrastructure" OR "green infrastructure" OR "integrated coastal zone management " OR "integrated water resources management" OR "protected area management" OR "ecosystem-based management" OR "wetland restoration" OR "floodplain restoration" OR "building with nature" OR "natural infrastructure" OR "river management" OR "ecosystem services" OR "landscape restoration" OR "coastal management" OR "coastal protection")) **AND**

(PUBYEAR > 1990) AND NOT TITLE-ABS-KEY ("alternative medicine" OR "childhood development" OR "cleft lip" OR "e. coli" OR "food safety" OR "machine learning" OR "mental illness" OR "renewable power" OR "search and rescue" OR "stress management" OR "technological disaster" OR "carbon credit" OR abusive OR ageing OR aging OR alcohol OR Alzheimer OR anaerobic OR antibiotic OR antidepressant OR anxiety OR arts OR autoreceptor OR biology OR cancer OR cardiovascular OR caribou OR circumcision OR coal OR compost OR consumer OR contaminat* OR customer OR dairy OR dance OR dementia OR depression OR diabetes OR diamorphine OR diet OR dietary OR digestates OR disease OR drug OR electricity OR electromagnetic OR emergency OR energy OR entrepreneurship OR evacuation OR e-waste OR exercise OR fracking OR fukushima OR garbage OR hernia OR hiv OR hunting OR infant OR influenza OR injury OR invertebrate OR macaque OR medical OR medication OR metabolic OR mice OR microbial OR milk OR mine OR myopia OR newborn OR nuclear OR nurse OR oil OR oxytocin OR pain OR particulate OR patient OR pediatric OR pension OR pesticide OR petrochemical OR phenotype OR phosphorus OR physician OR physiological OR poaching OR prenatal OR prophylaxis OR psychiatric OR psychosis OR "public housing" OR radiation OR radon OR railway OR resuscitat* OR robot OR rodent OR sarcoma OR sexual OR sleep OR stutter OR suicide OR surgeon OR surgical OR symptom OR terrorism OR terrorist OR thermoplastic OR ticks OR trpm2 OR UAV OR vaccine) **AND**

(LIMIT-TO (SUBJAREA,"ENVI") OR LIMIT-TO (SUBJAREA,"SOCI") OR LIMIT-TO (SUBJAREA,"AGRI") OR LIMIT-TO (SUBJAREA,"ENGI") OR LIMIT-TO (SUBJAREA,"EART") OR LIMIT-TO (SUBJAREA,"PSYC") OR LIMIT-TO (SUBJAREA,"ECON") OR LIMIT-TO (SUBJAREA,"ARTS") OR LIMIT-TO (SUBJAREA,"ENER") OR LIMIT-TO (SUBJAREA,"DECI") OR LIMIT-TO (SUBJAREA,"MULT"))

AND (LIMIT-TO (DOCTYPE,"ar") OR LIMIT-TO (DOCTYPE,"ch")) **AND**

(LIMIT-TO (LANGUAGE,"English") OR LIMIT-TO (LANGUAGE,"Spanish") OR LIMIT-TO (LANGUAGE,"French") OR LIMIT-TO (LANGUAGE,"German") OR LIMIT-TO (LANGUAGE,"Portuguese"))

Text S2. 97 articles and 2 book chapters included in the review.

1. Abbas, A., T. Amjath-Babu, H. Kächele, and K. Müller. 2016. Participatory adaptation to climate extremes: an assessment of households' willingness to contribute labor for flood risk mitigation in Pakistan. *Journal of Water and Climate Change* 7. International Water Association: 621–636.

2. Badola, R., and S. A. Hussain. 2005. Valuing ecosystem functions: an empirical study on the storm protection function of Bhitarkanika mangrove ecosystem, India. *Environmental Conservation* 32. Cambridge University Press: 85–92.

3. Badola, R., S. Barthwal, and S. A. Hussain. 2011. Attitudes of local communities towards conservation of mangrove forests: A case study from the east coast of India. *Estuarine, Coastal and Shelf Science* 96. Elsevier: 188–196.

4. Barbier, E. B. 2006. Natural barriers to natural disasters: replanting mangroves after the tsunami. *Frontiers in Ecology and the Environment* 4. Wiley Online Library: 124–131.

5. Barthélémy, C., and G. Armani. 2015. A comparison of social processes at three sites of the F rench Rhone River subjected to ecological restoration. *Freshwater Biology* 60. Wiley Online

Library: 1208–1220.

6. Beery, T. 2018. Engaging the Private Homeowner: Linking Climate Change and Green Stormwater Infrastructure. *Sustainability* 10. Multidisciplinary Digital Publishing Institute: 4791.

7. Bihari, M., and R. Ryan. 2012. Influence of social capital on community preparedness for wildfires. *Landscape and Urban Planning* 106. Elsevier: 253–261.

8. Biswas, S. R., A. U. Mallik, J. K. Choudhury, and A. Nishat. 2009. A unified framework for the restoration of Southeast Asian mangroves—bridging ecology, society and economics. *Wetlands Ecology and Management* 17. Springer: 365–383.

9. Bostick, T. P., T. H. Holzer, and S. Sarkani. 2017. Enabling stakeholder involvement in coastal disaster resilience planning. *Risk analysis* 37. Wiley Online Library: 1181–1200.

10. Boyer-Villemaire, U., P. Bernatchez, J. Benavente, and J. A. G. Cooper. 2014. Quantifying community's functional awareness of coastal changes and hazards from citizen perception analysis in Canada, UK and Spain. *Ocean \& coastal management* 93. Elsevier: 106–120.

11. Brandolini, S. M. D., and M. Disegna. 2015. ICZM and WTP of stakeholders for beach conservation: policymaking suggestions from an Italian case study. *Tourism Economics* 21. SAGE Publications Sage UK: London, England: 601–628.

12. Brink, E., and C. Wamsler. 2019. Citizen engagement in climate adaptation surveyed: The role of values, worldviews, gender and place. *Journal of cleaner production* 209. Elsevier: 1342–1353.

13. Bubeck, P., W. Botzen, L. Suu, and J. Aerts. 2012. Do flood risk perceptions provide useful insights for flood risk management? Findings from central Vietnam. *Journal of flood risk management* 5. Wiley Online Library: 295–302.

14. Buchecker, M., S. Menzel, and R. Home. 2013. How much does participatory flood management contribute to stakeholders' social capacity building? Empirical findings based on a triangulation of three evaluation approaches. *Natural Hazards and Earth System Sciences* 13. Copernicus GmbH: 1427–1444.

15. Buchecker, M., D. M. Ogasa, and E. Maidl. 2015. How well do the wider public accept integrated flood risk management? An empirical study in two Swiss Alpine valleys. *Environmental science \& policy* 55. Elsevier: 309–317.

16. Buijs, A. E. 2009. Public support for river restoration. A mixed-method study into local residents' support for and framing of river management and ecological restoration in the Dutch floodplains. *Journal of Environmental management* 90. Elsevier: 2680–2689.

17. Calvello, M., M. N. Papa, J. Pratschke, and M. N. Crescenzo. 2016. Landslide risk perception: a case study in Southern Italy. *Landslides* 13. Springer: 349–360.

18. Carro, I., L. Seijo, G. J. Nagy, X. Lagos, and O. Gutiérrez. 2018. Building capacity on ecosystem-based adaptation strategy to cope with extreme events and sea-level rise on the Uruguayan coast. *International Journal of Climate Change Strategies and Management* 10. Emerald Publishing Limited: 504–522.

19. Chan, F. K. S., O. A. Adekola, C. N. Ng, G. Mitchell, and A. T. McDonald. 2013. Coastal flood-risk management practice in Tai O, a town in Hong Kong. *Environmental Practice* 15. Cambridge University Press: 201–219.

20. Chen, C., Y. Wang, and J. Jia. 2018. Public perceptions of ecosystem services and preferences for design scenarios of the flooded bank along the Three Gorges Reservoir: Implications for sustainable management of novel ecosystems. *Urban Forestry \& Urban Greening* 34. Elsevier: 196–204.

21. Chou, R.-J. 2016. Achieving successful river restoration in dense urban areas: Lessons from Taiwan. *Sustainability* 8. Multidisciplinary Digital Publishing Institute: 1159.

22. Chowdhury, M. R. 2002. The Impact of Greater Dhaka Flood Protection Project'(GDFPP) on Local Living Environment-The Attitude of the Floodplain Residents. *Natural hazards* 29. Springer: 309–324.

23. Christianson, A., T. K. McGee, and L. L'Hirondelle. 2013. How historic and current wildfire experiences in an Aboriginal community influence mitigation preferences. *International Journal of Wildland Fire* 22. CSIRO: 527–536.

24. Damastuti, E., and R. de Groot. 2017. Effectiveness of community-based mangrove management for sustainable resource use and livelihood support: A case study of four villages in Central Java, Indonesia. *Journal of environmental management* 203. Elsevier: 510–521.

25. Davenport, M. A., C. A. Bridges, J. C. Mangun, A. D. Carver, K. W. Williard, and E. O. Jones. 2010. Building local community commitment to wetlands restoration: A case study of the Cache River wetlands in southern Illinois, USA. *Environmental management* 45. Springer: 711–722.

26. Davis, G., and K. Cole. 2004. Community involvement in coast protection at Lyme Regis, UK. In *Proceedings of the Institution of Civil Engineers-Municipal Engineer*, 158:17–22.

27. Dhakal, K. P., and L. R. Chevalier. 2017. Managing urban stormwater for urban sustainability: Barriers and policy solutions for green infrastructure application. *Journal of environmental management* 203: 171–181. doi:10.1016/j.jenvman.2017.07.065.

28. Ding, L., X. Ren, R. Gu, and Y. Che. 2019. Implementation of the "sponge city" development plan in China: An evaluation of public willingness to pay for the life-cycle maintenance of its facilities. *Cities* 93. Elsevier: 13–30.

29. Drake, B., J. C. Smart, M. Termansen, and K. Hubacek. 2013. Public preferences for production of local and global ecosystem services. *Regional environmental change* 13. Springer: 649–659.

30. Duan, J., Y. Wang, C. Fan, B. Xia, and R. de Groot. 2018. Perception of Urban Environmental Risks and the Effects of Urban Green Infrastructures (UGIs) on Human Well-being in Four Public Green Spaces of Guangzhou, China. *Environmental management* 62. Springer: 500–517.

31. Esteves, L. S., and K. Thomas. 2014. Managed realignment in practice in the UK: results from two independent surveys. *Journal of Coastal Research* 70. BioOne: 407–414.

32. Evans, A. J., B. Garrod, L. B. Firth, S. J. Hawkins, E. S. Morris-Webb, H. Goudge, and P. J. Moore. 2017. Stakeholder priorities for multi-functional coastal defence developments and steps to

effective implementation. *Marine Policy* 75. Elsevier: 143–155.

33. Everett, G., and J. Lamond. 2014. A conceptual framework for understanding behaviours and attitudes around "Blue-Green"approaches to Flood-Risk Management. *Flood Recovery, Innovation and Response IV* 184. WIT Press Southampton, UK: 101.

34. Everett, G., and J. E. Lamond. 2018. Considering the value of community engagement for (co-) producing blue-green infrastructure. *WIT Transactions on The Built Environment* 184. WIT Press: 1–13.

35. Everett, G., J. Lamond, A. T. Morzillo, A. M. Matsler, and F. K. S. Chan. 2018. Delivering Green Streets: an exploration of changing perceptions and behaviours over time around bioswales in Portland, Oregon. *Journal of Flood Risk Management* 11. Wiley Online Library: S973–S985.

36. Figueiredo, E., S. Valente, C. Coelho, and L. Pinho. 2009. Coping with risk: analysis on the importance of integrating social perceptions on flood risk into management mechanisms-the case of the municipality of Águeda, Portugal. *Journal of Risk Research* 12. Taylor \& Francis: 581–602.

37. Fordham, M., S. Tunstall, and E. Penning-Rowsell. 1991. Choice and preference in the Thames floodplain: the beginnings of a participatory approach? *Landscape and Urban Planning* 20. Elsevier: 183–187.

38. Fuchs, S., K. Karagiorgos, K. Kitikidou, F. Maris, S. Paparrizos, and T. Thaler. 2017. Flood risk perception and adaptation capacity: A contribution to the socio-hydrology debate. *Hydrology and Earth System Sciences* 21. Copernicus GmbH: 3183–3198.

39. Geaves, L. H., and E. C. Penning-Rowsell. 2015. Flood risk management as a public or a private good, and the implications for stakeholder engagement. *Environmental Science \& Policy* 55. Elsevier: 281–291.

40. Ghanbarpour, M. R., M. M. Saravi, and S. Salimi. 2014. Floodplain inundation analysis combined with contingent valuation: implications for sustainable flood risk management. *Water resources management* 28. Springer: 2491–2505.

41. Gilman, E., and J. Ellison. 2007. Efficacy of alternative low-cost approaches to mangrove restoration, American Samoa. *Estuaries and Coasts* 30. Springer: 641–651.

42. Godschalk, D., S. Brody, and R. Burby. 2003. Public Participation in Natural Hazard Mitigation Policy Formation: Challenges for Comprehensive Planning. *Journal of Environmental Planning and Management* 46: 733–754.

43. Goeldner-Gianella, L., F. Bertrand, A. Oiry, and D. Grancher. 2015. Depolderisation policy against coastal flooding and social acceptability on the French Atlantic coast: The case of the Arcachon Bay. *Ocean \& Coastal Management* 116. Elsevier: 98–107.

44. Gray, J. D. E., K. O'Neill, and Z. Qiu. 2017. Coastal residents' perceptions of the function of and relationship between engineered and natural infrastructure for coastal hazard mitigation. *Ocean \& Coastal Management* 146. Elsevier: 144–156.

45. De Groot, M., and W. T. de Groot. 2009. "Room for river" measures and public visions in the Netherlands: A survey on river perceptions among riverside residents. *Water Resources Research*

45. Wiley Online Library.

46. Herringshaw, C. J., J. R. Thompson, and T. W. Stewart. 2010. Learning about restoration of urban ecosystems: a case study integrating public participation, stormwater management, and ecological research. *Urban ecosystems* 13. Springer: 535–562.

47. Van den Hoek, R., M. Brugnach, J. Mulder, and A. Hoekstra. 2014. Uncovering the origin of ambiguity in nature-inclusive flood infrastructure projects. *Ecology and society* 19. The Resilience Alliance.

48. Holcombe, E. A., and M. G. Anderson. 2010. Implementation of community-based landslide hazard mitigation measures: the role of stakeholder engagement in “sustainable”project scale-up. *Sustainable Development* 18. Wiley Online Library: 331–349.

49. Holcombe, E. A., E. Berg, S. Smith, M. G. Anderson, and N. Holm-Nielsen. 2018. Does participation lead to ongoing infrastructure maintenance? Evidence from Caribbean landslide mitigation projects. *The Journal of Development Studies* 54. Taylor \& Francis: 1374–1391.

50. Holstead, K., W. Kenyon, J. Rouillard, J. Hopkins, and C. Galán-Diaz. 2017. Natural flood management from the farmer’s perspective: criteria that affect uptake. *Journal of Flood Risk Management* 10. Wiley Online Library: 205–218.

51. Hoque, M. M., and M. A. Siddique. 1995. Flood control projects in Bangladesh: reasons for failure and recommendations for improvement. *Disasters* 19: 260–3.

52. Houston, D., W. Cheung, V. Basolo, D. Feldman, R. Matthew, B. F. Sanders, B. Karlin, J. E. Schubert, et al. 2019. The Influence of Hazard Maps and Trust of Flood Controls on Coastal Flood Spatial Awareness and Risk Perception. *Environment and Behavior* 51. SAGE Publications Sage CA: Los Angeles, CA: 347–375.

53. Howgate, O. R., and W. Kenyon. 2009. Community cooperation with natural flood management: a case study in the Scottish Borders. *Area* 41. Wiley Online Library: 329–340.

54. Iftekhar, M., and T. Takama. 2008. Perceptions of biodiversity, environmental services, and conservation of planted mangroves: a case study on Nijhum Dwip Island, Bangladesh. *Wetlands Ecology and Management* 16. Springer: 119–137.

55. Jones, N., S. Koukoulas, J. Clark, K. Evangelinos, P. Dimitrakopoulos, M. Eftihidou, A. Koliou, M. Mpalaska, et al. 2014. Social capital and citizen perceptions of coastal management for tackling climate change impacts in Greece. *Regional environmental change* 14. Springer: 1083–1093.

56. Kenyon, W. 2007. Evaluating flood risk management options in Scotland: A participant-led multi-criteria approach. *Ecological Economics* 64: 70–81.

57. Khew, Y. T. J., M. P. Jarzebski, F. Dyah, R. San Carlos, J. Gu, M. Esteban, R. Aránguiz, and T. Akiyama. 2015. Assessment of social perception on the contribution of hard-infrastructure for tsunami mitigation to coastal community resilience after the 2010 tsunami: Greater Concepcion area, Chile. *International journal of disaster risk reduction* 13. Elsevier: 324–333.

58. Kienker, S., R. Coleman, R. Morris, P. Steinberg, B. Bollard, R. Jarvis, K. Alexander, and E. Strain. 2018. Bringing harbours alive: assessing the importance of eco-engineered coastal infrastructure

for different stakeholders and cities. *Marine Policy* 94. Elsevier: 238–246.

59. Kim, T.-G., and D. R. Petrolia. 2013. Public perceptions of wetland restoration benefits in Louisiana. *ICES Journal of Marine Science* 70. Oxford University Press: 1045–1054.

60. Koutrakis, E., A. Sapounidis, S. Marzetti, V. Marin, S. Roussel, S. Martino, M. Fabiano, C. Paoli, et al. 2011. ICZM and coastal defence perception by beach users: lessons from the Mediterranean coastal area. *Ocean \& coastal management* 54. Elsevier: 821–830.

61. Kuo, Y.-L., C.-C. Chang, and H.-C. Li. 2015. Lulling effect of public flood protection: Case of Benhe community in Kaohsiung during Typhoon Fanapi. *Natural Hazards Review* 17. American Society of Civil Engineers: 05015003.

62. Lara, A., D. Sauri, A. Ribas, and D. Pavón. 2010. Social perceptions of floods and flood management in a Mediterranean area (Costa Brava, Spain). *Natural Hazards and Earth System Sciences* 10. Citeseer: 2081.

63. McCarthy, S., and E. Penning-Rowsell. 2008. Public attitudes to “community-based” small-scale flood risk reduction measures in England: A case study in the Lower Thames catchment. In *Hazards and the Built Environment*, 168–189. Routledge.

64. McGee, T. K. 2007. Urban residents’ approval of management measures to mitigate wildland-urban interface fire risks in Edmonton, Canada. *Landscape and Urban Planning* 82. Elsevier: 247–256.

65. Metzger, A., and J. Linton. 2017. Des inondations barrées? La représentation des vulnérabilités en aval des barrages réservoirs. *Espace populations sociétés. Space populations societies*. Université des Sciences et Technologies de Lille.

66. Miller, S. M., and F. A. Montalto. 2019. Stakeholder perceptions of the ecosystem services provided by Green Infrastructure in New York City. *Ecosystem Services* 37. Elsevier: 100928.

67. Myatt, L. B., M. D. Scrimshaw, and J. N. Lester. 2003a. Public perceptions and attitudes towards a forthcoming managed realignment scheme: Freiston Shore, Lincolnshire, UK. *Ocean & Coastal Management* 46. Elsevier: 565–582.

68. Myatt, L. B., M. D. Scrimshaw, and J. N. Lester. 2003b. Public perceptions and attitudes towards an established managed realignment scheme: Orplands, Essex, UK. *Journal of environmental management* 68: 173–81.

69. Myatt-Bell, L. B., M. D. Scrimshaw, J. N. Lester, and J. S. Potts. 2002. Public perception of managed realignment: Brancaster West Marsh, North Norfolk, UK. *Marine Policy* 26: 45–57.

70. Neef, A., P. Elstner, and I. Schad. 2014. The interplay between collective action, individual strategies and state intervention in mitigating flood disasters in the uplands of North Thailand and Northwest Vietnam. In *Risks and Conflicts: Local Responses to Natural Disasters*, 109–130. Emerald Group Publishing Limited.

71. Nguyen, T., N. Van Tam, K. E. Parnell, and others. 2015. Community perspectives on an internationally funded mangrove restoration project: Kien Giang province, Vietnam. *Ocean &*

coastal management 119. Elsevier: 146–154.

72. On-prom, S. 2014. Community-based mangrove forest management in Thailand: key lesson learned for environmental risk management. In *Sustainable Living with Environmental Risks*, 87–96. Springer.

73. Otto, A., A. Hornberg, and A. Thieken. 2018. Local controversies of flood risk reduction measures in Germany. An explorative overview and recent insights. *Journal of Flood Risk Management* 11. Wiley Online Library: S382–S394.

74. Pueyo-Ros, J., A. Ribas, and R. M. Fraguell. 2018. A cultural approach to wetlands restoration to assess its public acceptance. *Restoration Ecology* 27. Wiley Online Library: 626–637.

75. Rambonilaza, T., O. Joalland, and E. Brahic. 2016. Landowner's perception of flood risk and preventive actions in estuarine environment: An empirical investigation. *Journal of environmental management* 180. Elsevier: 272–279.

76. Rasid, H., S. Shuncai, Y. Xiubo, and Z. Chen. 1996. Structural vs Non-structural Flood-alleviation Measures in the Yangtze Delta: A Pilot Survey of Floodplain Residents' Preferences. *Disasters* 20. Wiley Online Library: 93–110.

77. Reilly, K., J. Adamowski, and K. John. 2018. Participatory mapping of ecosystem services to understand stakeholders' perceptions of the future of the Mactaquac Dam, Canada. *Ecosystem services* 30. Elsevier: 107–123.

78. Reynaud, A., D. Lanzanova, C. Liquete, and B. Grizzetti. 2017. Going green? Ex-post valuation of a multipurpose water infrastructure in Northern Italy. *Ecosystem services* 27. Elsevier: 70–81.

79. Roca, E., and M. Villares. 2012. Public perceptions of managed realignment strategies: the case study of the Ebro Delta in the Mediterranean basin. *Ocean & Coastal Management* 60. Elsevier: 38–47.

80. Ryan, R. L., and M. B. Wamsley. 2008. Public perceptions of wildfire risk and forest management in the Central Pine Barrens of Long Island (USA). *The Australasian Journal of Disaster and Trauma Studies* 2: 1–16.

81. Saengsupavanich, C. 2012. Detached breakwaters: communities' preferences for sustainable coastal protection. *Journal of environmental management* 115: 106–13. doi:10.1016/j.jenvman.2012.11.029.

82. Saengsupavanich, C. 2013. Erosion protection options of a muddy coastline in Thailand: stakeholders' shared responsibilities. *Ocean \& coastal management* 83. Elsevier: 81–90.

83. Santoro, S., I. Pluchinotta, A. Pagano, P. Pengal, B. Cokan, and R. Giordano. 2019. Assessing stakeholders' risk perception to promote Nature Based Solutions as flood protection strategies: The case of the Glin\vs\vcica river (Slovenia). *Science of the Total Environment* 655. Elsevier: 188–201.

84. Scally, J., and G. Wescott. 2011. Perceptions of climate change and adaptation responses in a local community: The Barwon Estuary Complex, Victoria. *Australian Geographer* 42. Taylor \& Francis:

387–401.

85. Schaich, H. 2009. Local residents' perceptions of floodplain restoration measures in Luxembourg's Syr Valley. *Landscape and Urban Planning* 93. Elsevier: 20–30.

86. Schernewski, G., J. Schumacher, E. Weisner, and L. Donges. 2017. A combined coastal protection, realignment and wetland restoration scheme in the southern Baltic: planning process, public information and participation. *Journal of coastal conservation* 22. Springer: 533–547.

87. Schmidt, L., C. Gomes, S. Guerreiro, and T. O'Riordan. 2013. Are we all on the same boat? The challenge of adaptation facing Portuguese coastal communities: Risk perception, trust-building and genuine participation. *Land Use Policy* 38. Elsevier: 355–365.

88. Scholte, S. S., M. Todorova, A. J. van Teeffelen, and P. H. Verburg. 2016. Public support for wetland restoration: what is the link with ecosystem service values? *Wetlands* 36. Springer: 467–481.

89. Tanaka, N., K. Jinadasa, M. Mowjood, and M. Fasly. 2011. Coastal vegetation planting projects for tsunami disaster mitigation: effectiveness evaluation of new establishments. *Landscape and ecological engineering* 7. Springer: 127–135.

90. Tompkins, E. L., R. Few, and K. Brown. 2008. Scenario-based stakeholder engagement: incorporating stakeholders preferences into coastal planning for climate change. *Journal of environmental management* 88. Elsevier: 1580–1592.

91. Touili, N., J. Baztan, J.-P. Vanderlinden, I. O. Kane, P. Diaz-Simal, and L. Pietrantoni. 2014. Public perception of engineering-based coastal flooding and erosion risk mitigation options: Lessons from three European coastal settings. *Coastal Engineering* 87: 205 – 209. doi:https://doi.org/10.1016/j.coastaleng.2014.01.004.

92. Trialfhianty, T. I., and Suadi. 2017. The role of the community in supporting coral reef restoration in Pemuteran, Bali, Indonesia. *Journal of coastal conservation* 21. Springer: 873–882.

93. Triyanti, A., M. Bavinck, J. Gupta, and M. A. Marfai. 2017. Social capital, interactive governance and coastal protection: The effectiveness of mangrove ecosystem-based strategies in promoting inclusive development in Demak, Indonesia. *Ocean & coastal management* 150. Elsevier: 3–11.

94. Vávra, J., M. Lapka, E. Cudlinová, and Z. Dvovráková-Livsková. 2017. Local perception of floods in the Czech Republic and recent changes in state flood management strategies. *Journal of Flood Risk Management* 10. Wiley Online Library: 238–252.

95. Verbrugge, L. N., W. Ganzevoort, J. M. Fliervoet, K. Panten, and R. J. van den Born. 2017. Implementing participatory monitoring in river management: The role of stakeholders' perspectives and incentives. *Journal of environmental management* 195. Elsevier: 62–69.

96. Wang, H.-W., A. Dodd, P.-H. Kuo, and B. LePage. 2018. Science as a Bridge in Communicating Needs and Implementing Changes towards Wetland Conservation in Taiwan. *Wetlands* 38. Springer: 1223–1232.

97. Wang, Y., M. Sun, and B. Song. 2017. Public perceptions of and willingness to pay for sponge city initiatives in China. *Resources, Conservation and Recycling* 122. Elsevier: 11–20.

98. Wedawatta, G., U. Kulatunga, D. Amaratunga, and A. Parvez. 2016. Disaster risk reduction infrastructure requirements for South-Western Bangladesh: Perspectives of local communities. *Built Environment Project and Asset Management* 6. Emerald Group Publishing Limited: 379–390.

99. Zhang, M., Y. Chen, and X. Wu. 2019. Resident preferences for augmented rainstorm disasters management strategies: The case of nanjing in china. *Environmental Hazards* 18. Taylor \& Francis: 78–92.
